# Supplementary material for: Rab2A-mediated Golgi-lipid droplet interactions support very-low-density lipoprotein secretion in hepatocytes
Source: EMBO J. 2024 Nov 4;43(24):6383–409. doi: 10.1038/s44318-024-00288-x (PMC11649929; doi:10.1038/s44318-024-00288-x)
Supplement: Supplementary file 1 — Appendix [file 44318_2024_288_MOESM1_ESM.pdf]

Rab2A-mediated Golgi-Lipid droplet interactions support very-low-density lipoprotein secretion in hepatocytes.

APPENDIX

Appendix Figure S1: Generation and analysis of the *Rab2a* hepatocytes-specific knockout mice....p2

Appendix Figure S2: Quantifying the lipid levels and apolipoproteins expression levels in the livers of Flox and LCK mice**...**p4

Appendix Figure S3: Evaluating the rates of lipid uptake in Flox and LCK mice....p6

Appendix Figure S4: Quality verification of sucrose density gradient centrifugation assay**...**p7

Appendix Figure S5: GTP-bound Rab2A predominantly locates to Golgi apparatus**...**p9

Appendix Figure S6: Evaluating the morphological features of the Golgi apparatus in Flox and LCK hepatocytes....p10

Appendix Figure S7: An illustrative depiction on lipid droplets (LDs) pulldown assay in Huh7 cells and livers were provided....p11

Appendix Figure S8: Rab2A binds with LD-localized protein, 17-beta-hydroxysteroid dehydrogenase 13 (HSD17B13)....p13

Appendix Figure S9: Confirming the location of RCAS1 at Golgi apparatus....p14

Appendix Figure S10: Hepatic HSD17B13 deficiency mitigates very-low-density lipoprotein (VLDL) secretion**...**p15

Appendix Figure S11: Mapping the specific binding sites between Rab2A and HSD17B13.....p16

Appendix Figure S12: Fasted suppresses VLDL secretion.....p17

Appendix Figure S13: Quality verification of Golgi-Fractions.....p18

Appendix Table S1: List of 65 interacting proteins of Rab2A identified by mass spectrometry.....p19

Appendix Figure S1: Generation and analysis of the *Rab2a* hepatocytes-specific knockout mice.

(A) Schematic representation of the construction of *Rab2a*-flox mice (Flox) and *Rab2a* hepatocytes-specific knockout mice (LCK).

(B) Validation of the knockout specificity of Ras-related protein Rab-2A (Rab2A) in LCK mice compared with wild-type (WT) and Flox mice using Western blotting.

(C) The full gel blotting further proved the knockout specificity of Rab2A in LCK mice.

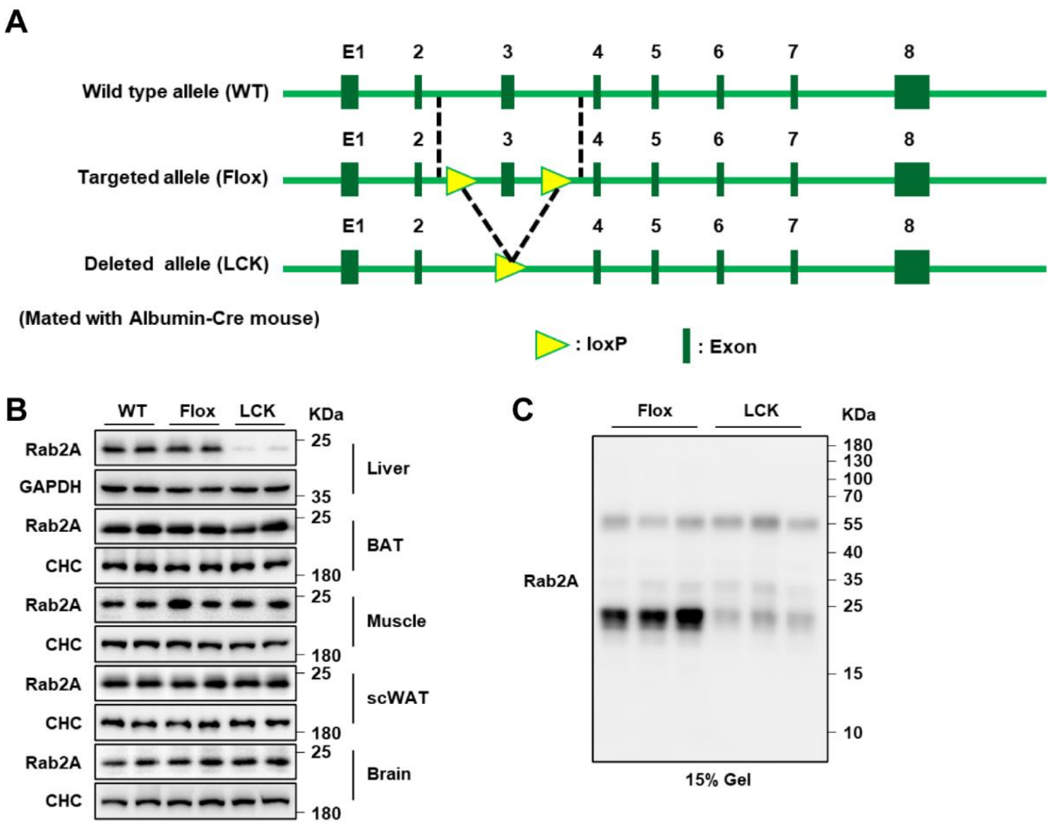

Appendix Figure S2: Quantifying the lipid levels and apolipoproteins expression levels in the livers of Flox and LCK mice.

(A, B) Quantification of triglycerides (TG) (**A**) and total cholesterol (TC) (**B**) levels in the livers (Male, n=5 mice per group).

(C, D) Assessment of the expression levels of lipoprotein synthesis-related proteins in the livers of mice under "Random feed" and "Fasted" conditions, including Apo B-100, Apo B-48, Apo-E, Apo-AI, Apo-CIII, Microsomal triglyceride transfer protein large subunit (MTP), Low-density lipoprotein receptor (LDLR), and CD36 (**C**), followed by grayscale quantification of the relevant proteins, with normalization to 1 for Flox samples (**D**) (Male, n=3 mice per group).

(E, F) Measurement of TG (**E**) and TC (**F**) levels in liver samples collected from mice fed with High-fat-high-cholesterol diet (HFHCD) for three months (Male, n=6 vs. 5 mice).

(G, H) Assessment of the expression levels of lipoprotein synthesis-related proteins in the livers of mice challenged with HFHCD, including Apo B-100, Apo B-48, Apo-AI, Apo-CIII and MTP (**G**), followed by grayscale quantification of the relevant proteins, with normalization to 1 for Flox samples (**H**) (Male, n=4 mice per group).

Data information: Data in (**A, B, D, E, F, H**) are presented as mean ± SEM. Circles in (**A, B, D, E, F, H**) correspond to individual mice. *P* values in (**A, B, D, E, F, H**) were determined using unpaired two-tailed Student's t-test. n.s. indicates no significant difference ($p > 0.05$), * indicates $p < 0.05$; ** indicates $p < 0.01$; *** indicates $p < 0.001$.

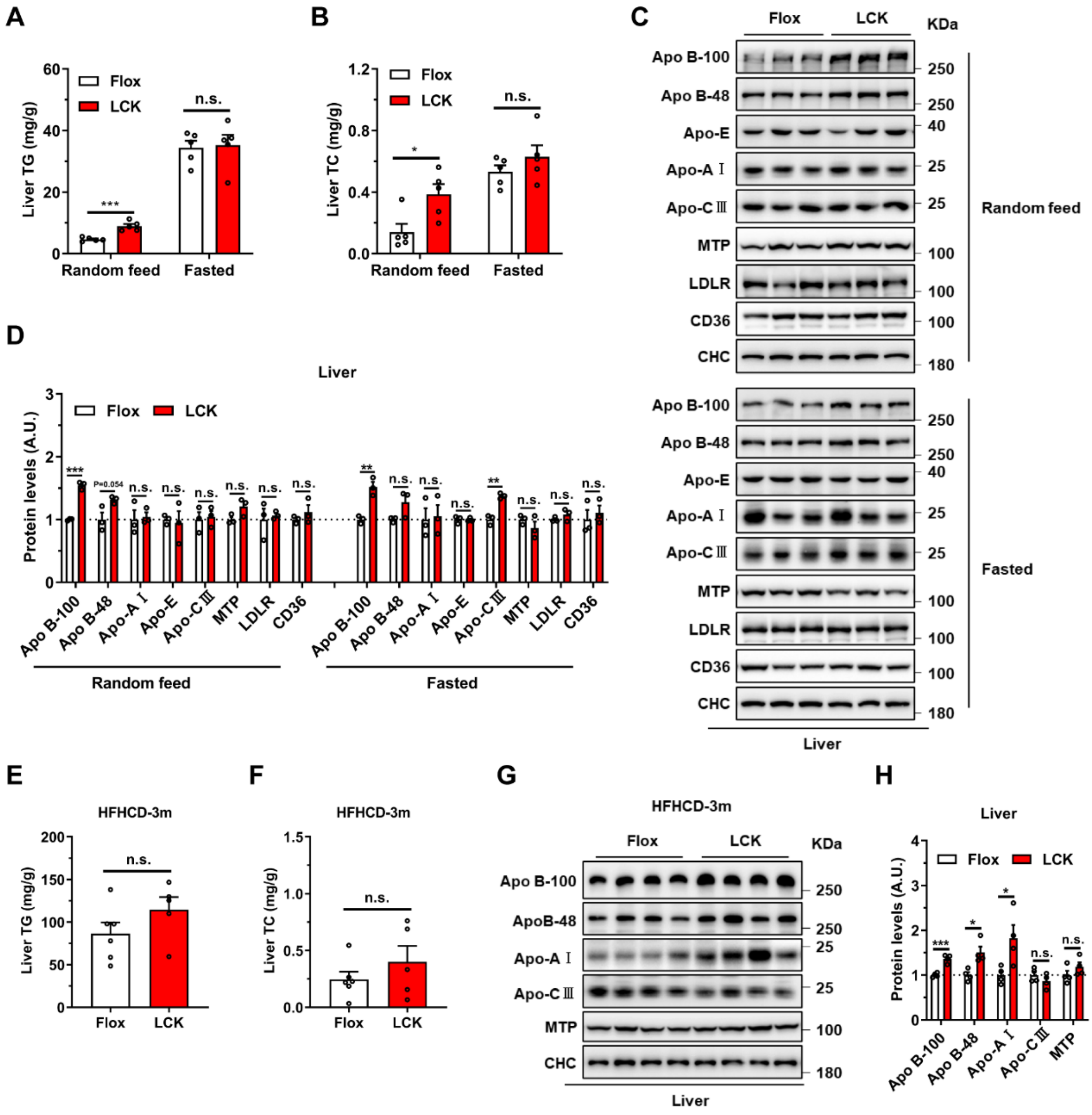

A
Liver TG (mg/g)
Flox
LCK
Random feed
Fasted
B
Liver TC (mg/g)
C
Flox
LCK
KDa
Apo B-100
Apo B-48
Apo-E
Apo-A Ⅰ
Apo-C Ⅲ
MTP
LDLR
CD36
CHC
Random feed
Fasted
Liver
D
Liver
Protein levels (A.U.)
E
HFHCD-3m
Liver TG (mg/g)
F
HFHCD-3m
Liver TC (mg/g)
G
HFHCD-3m
H
Liver
Protein levels (A.U.)

Appendix Figure S3: Evaluating the rates of lipid uptake in Flox and LCK mice.

(A) Fatty-acid absorption levels in Flox and LCK mice were evaluated through a lipid gavage assay. TG level in serum were quantified (Male, n=6 mice per group).

(B-C) Quantifying the rates of fatty acid absorption in primary hepatocytes isolated from Flox and LCK mice. The representative images were shown (**B**) and the rates were evaluated (n=71 vs. 96 cells) (**C**).

(D-H) LDLR knockout and LDLR/Rab2A double knockout mice were bred and then fed with WD (Western diet) for 2 months. Parameters in serum and liver samples were assessed (Male, n=7 vs. 10 mice), including protein levels of Rab2A and LDLR in the liver (**D**), serum TG level (**E**) and detailed distributions in lipoproteins (**G**), serum TC level (**F**) and detailed distributions in lipoproteins (**H**).

Data information: Data in (**A, C, E, F**) are presented as mean ± SEM. Circles in (**C**) correspond to individual primary hepatocyte. Circles in (**E, F**) correspond to individual mice. *P* value in (**A**) was determined using two-way ANOVA. *P* values in (**C, E, F**) were determined using unpaired two-tailed Student's t-test. n.s. indicates no significant difference ($p > 0.05$), ** indicates $p < 0.01$; *** indicates $p < 0.001$.

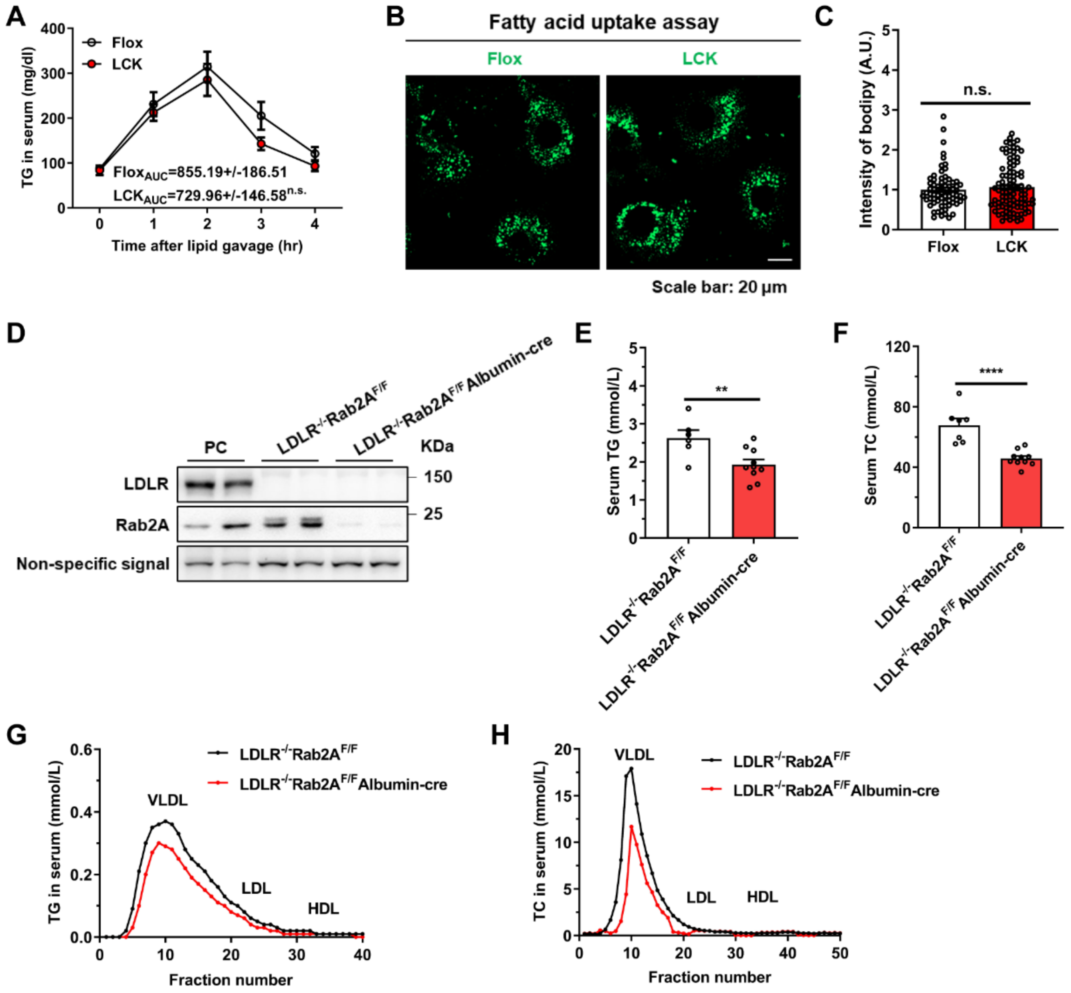
A
Flox
LCK
TG in serum (mg/dl)
Time after lipid gavage (hr)
FloxAUC=855.19+/-186.51
LCKAUC=729.96+/-146.58n.s.
B
Fatty acid uptake assay
Flox
LCK
Scale bar: 20 μm
C
Intensity of bodipy (A.U.)
n.s.
Flox
LCK
D
PC
LDLR-/-Rab2AF/F
LDLR-/-Rab2AF/F Albumin-cre
KDa
LDLR
Rab2A
Non-specific signal
150
25
E
Serum TG (mmol/L)
**
F
Serum TC (mmol/L)
****
G
TG in serum (mmol/L)
LDLR-/-Rab2AF/F
LDLR-/-Rab2AF/FAlbumin-cre
VLDL
LDL
HDL
Fraction number
H
TC in serum (mmol/L)
VLDL
LDL
HDL
Fraction number

Appendix Figure S4: Quality verification of sucrose density gradient centrifugation assay.

(A-B) Validation of the quality of sucrose density gradient centrifugation assay through western blotting, utilizing Golgin-97 and GM130 as Golgi markers, GRP-94 and PDI as ER markers, Perilipin-3 as LD marker, Actin as cytosol marker, and Lamin-B1 as nuclear marker.

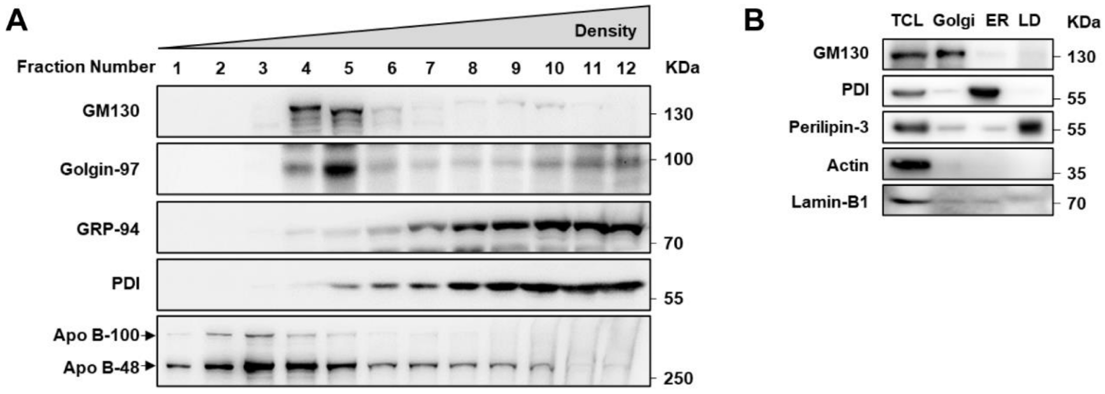

Appendix Figure S5: GTP-bound Rab2A predominantly locates to Golgi apparatus.

(A-B) Subcellular distribution of Rab2A (exogenously overexpressed Rab2A) was scrutinized in Huh7 cells via immunofluorescence, using GM130 as a Golgi marker, ERGIC-53 as an ERGIC marker, and KDEL labeling to identify the ER (**A**). The corresponding Pearson's R values were analyzed via Image J (n=17 vs. 15 vs. 17 cells) (**B**).

(C) Immunofluorescence analysis delineating the subcellular distribution of GTP-bound Rab2A and GDP-bound Rab2A, with GM130 serving as the marker of Golgi in Huh7 cells.

Data information: Data in (**B**) are presented as mean ± SEM. Circles in (**B**) correspond to individual Huh7 cell. *P* values in (**B**) were determined using unpaired two-tailed Student's t-test. *** indicates $p < 0.001$.

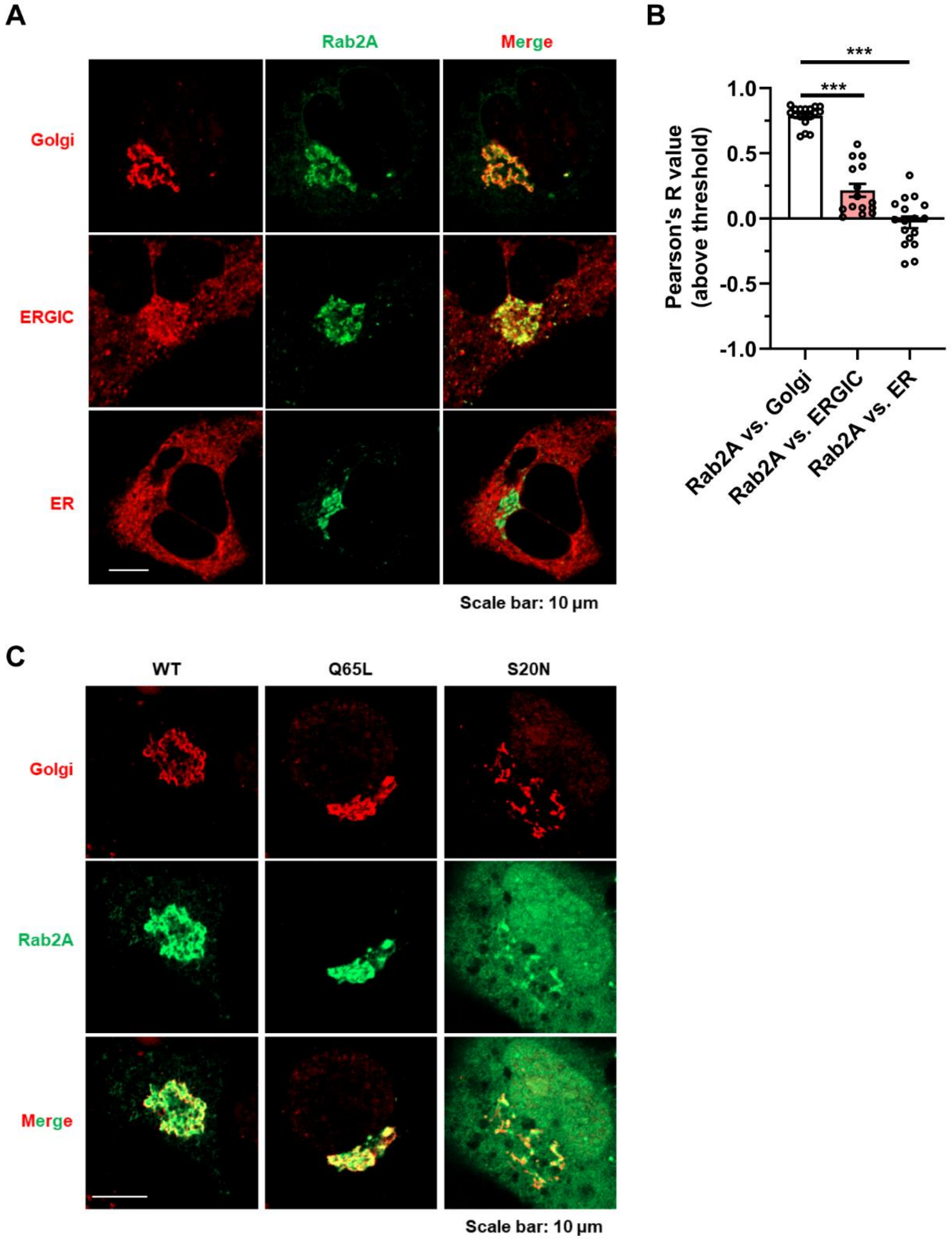
A
Rab2A
Merge
Golgi
ERGIC
ER
Scale bar: 10 μm
B
***
***
Pearson's R value (above threshold)
1.0
0.5
0.0
-0.5
-1.0
Rab2A vs. Golgi
Rab2A vs. ERGIC
Rab2A vs. ER
C
WT
Q65L
S20N
Golgi
Rab2A
Merge
Scale bar: 10 μm

Appendix Figure S6: Evaluating the morphological features of the Golgi apparatus in Flox and LCK hepatocytes.

(A-B) Assessment of Golgi apparatus morphology in primary hepatocytes from Flox and LCK mice via immunofluorescence, using Golgin-97 as the marker of Golgi, and DAPI for nuclear labeling. The represented images were shown (**A**), and Golgin-97-labeled spot area was quantified (n=32 vs. 19 cells) (**B**).

(C) Morphological features of the Golgi apparatus in primary hepatocytes from Flox and LCK mice were examined using transmission electron microscopy (TEM).

Data information: Data in (**B**) are presented as mean ± SEM. Circles in (**B**) correspond to individual primary hepatocyte. *P* value in (**B**) was determined using unpaired two-tailed Student's t-test. n.s. indicates no significant difference (p >0.05).

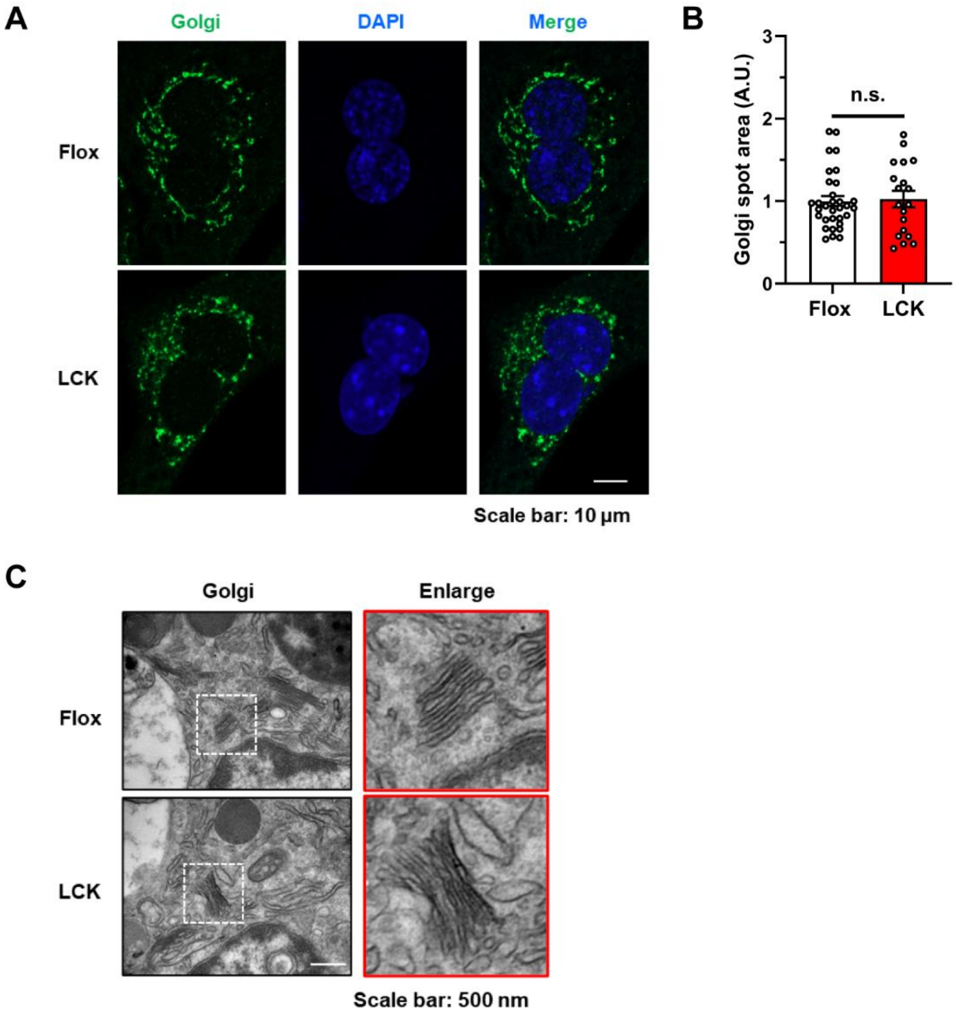

Appendix Figure S7: An illustrative depiction on lipid droplets (LDs) pulldown assay in primary hepatocytes and livers were provided.

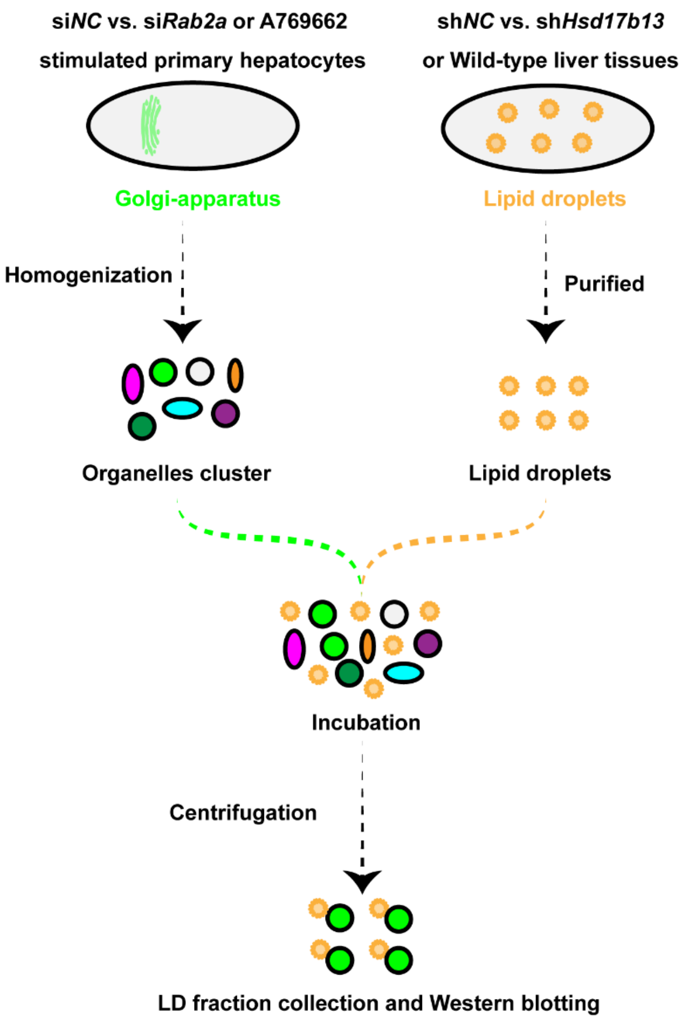

Appendix Figure S8: Rab2A binds with LD-localized protein, 17-beta-hydroxysteroid dehydrogenase 13 (HSD17B13).

(A) The critical role of Rab2A localization in the binding between Rab2A and HSD17B13 was analyzed in HEK293T cells through transfection with Rab2A mutation plasmids, followed by the MYC-affinity beads pulldown assay.

(B) Huh7 cells were transfected with wild-type and mutated Rab2A plasmids to investigate the roles of variant regions of Rab2A in localization via immunofluorescence (Golgi was labeled with RCAS1-BFP).

(C) Evaluating the roles of Rab2A variant regions in the binding between Rab2A and HSD17B13, HEK293T cells were transfected with corresponding Rab2A mutation plasmids, followed by MYC-affinity beads pulldown assay.

(D, E) The precise localization of HSD17B13 on LDs and its subsequent functions in the interaction between Rab2A and HSD17B13 were assessed in Huh7 cells and HEK293T cells via relevant plasmid transfection, and visualization by immunofluorescence (**D**) and Western blotting (**E**).

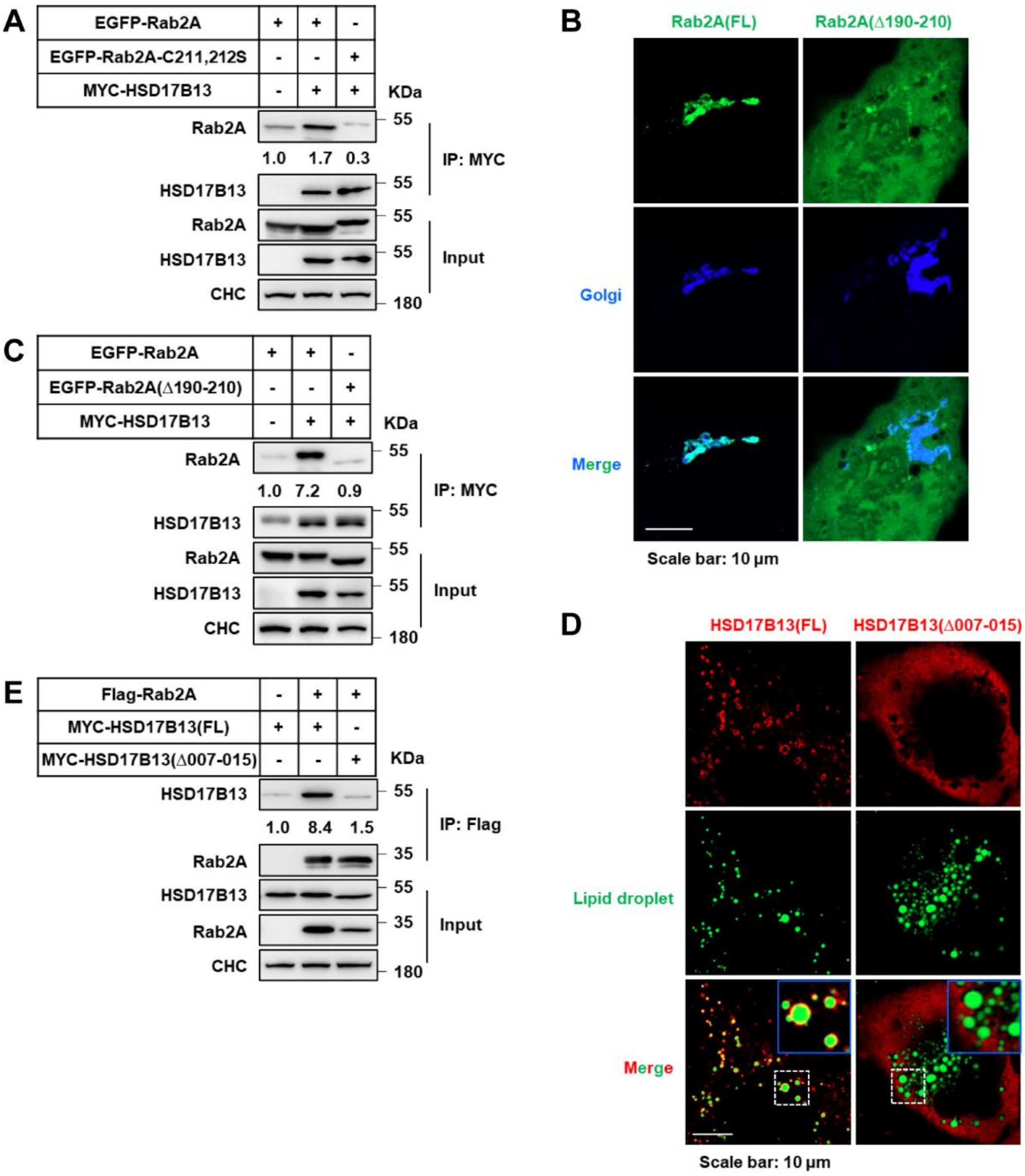
A
EGFP-Rab2A + + -
EGFP-Rab2A-C211,212S - - +
MYC-HSD17B13 - + +
KDa
Rab2A
55
1.0 1.7 0.3
IP: MYC
HSD17B13
Input
CHC
180
B
Rab2A(FL)
Rab2A(Δ190-210)
Golgi
Merge
Scale bar: 10 μm
C
EGFP-Rab2A + + -
EGFP-Rab2A(Δ190-210) - - +
MYC-HSD17B13 - + +
1.0 7.2 0.9
D
HSD17B13(FL)
HSD17B13(Δ007-015)
Lipid droplet
Merge
Scale bar: 10 μm
E
Flag-Rab2A - + +
MYC-HSD17B13(FL) + + -
MYC-HSD17B13(Δ007-015) - - +
HSD17B13
1.0 8.4 1.5
IP: Flag
Rab2A
35

Appendix Figure S9: Confirming the location of RCAS1 at Golgi apparatus.

(A) Huh7 cells overexpressing RCAS1-BFP plasmids were stained with a GM130 primary antibody, and immunofluorescence was performed to identify the subcellular localization of RCAS1.

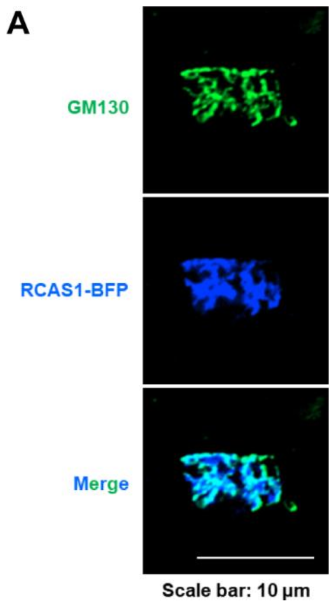

Appendix Figure S10: Hepatic HSD17B13 deficiency mitigates very-low-density lipoprotein (VLDL) secretion.

(A-E) In vivo inhibition of HSD17B13 was achieved in wild-type mice livers using adeno-associated virus serotype 2/8 (AAV2/8)-shRNA virus (Male, n=7 vs. 5 mice). Several experiments were conducted to explore the impact of HSD17B13 on VLDL secretion, as illustrated (**A**). TG and TC levels in liver (**B, C**), and lipid secretion after tyloxapol injection (**D, E**) were systematically assessed.

Data information: Data in (**B, C, D**) are presented as mean ± SEM. Circles in (**B, C**) correspond to individual mice. *P* values in (**B, C**) were determined using unpaired two-tailed Student's t-test. *P* value in (**D**) was determined using two-way ANOVA. n.s. indicates no significant difference ($p > 0.05$); * indicates $p < 0.05$; *** indicates $p < 0.001$.

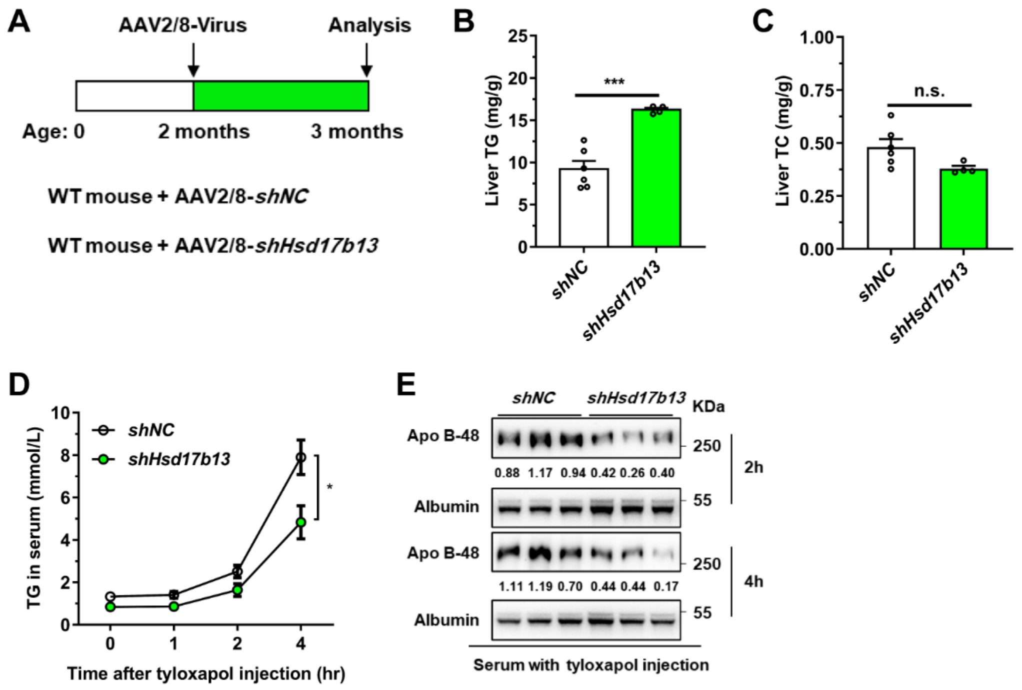

Appendix Figure S11: Mapping the specific binding sites between Rab2A and HSD17B13.

(A-F) The precise binding sites within Rab2A were delineated in HEK293T cells transfected with mutated Rab2A plasmids. The binding efficiency was assessed step-by-step using Flag-affinity or MYC-affinity beads pulldown assays.

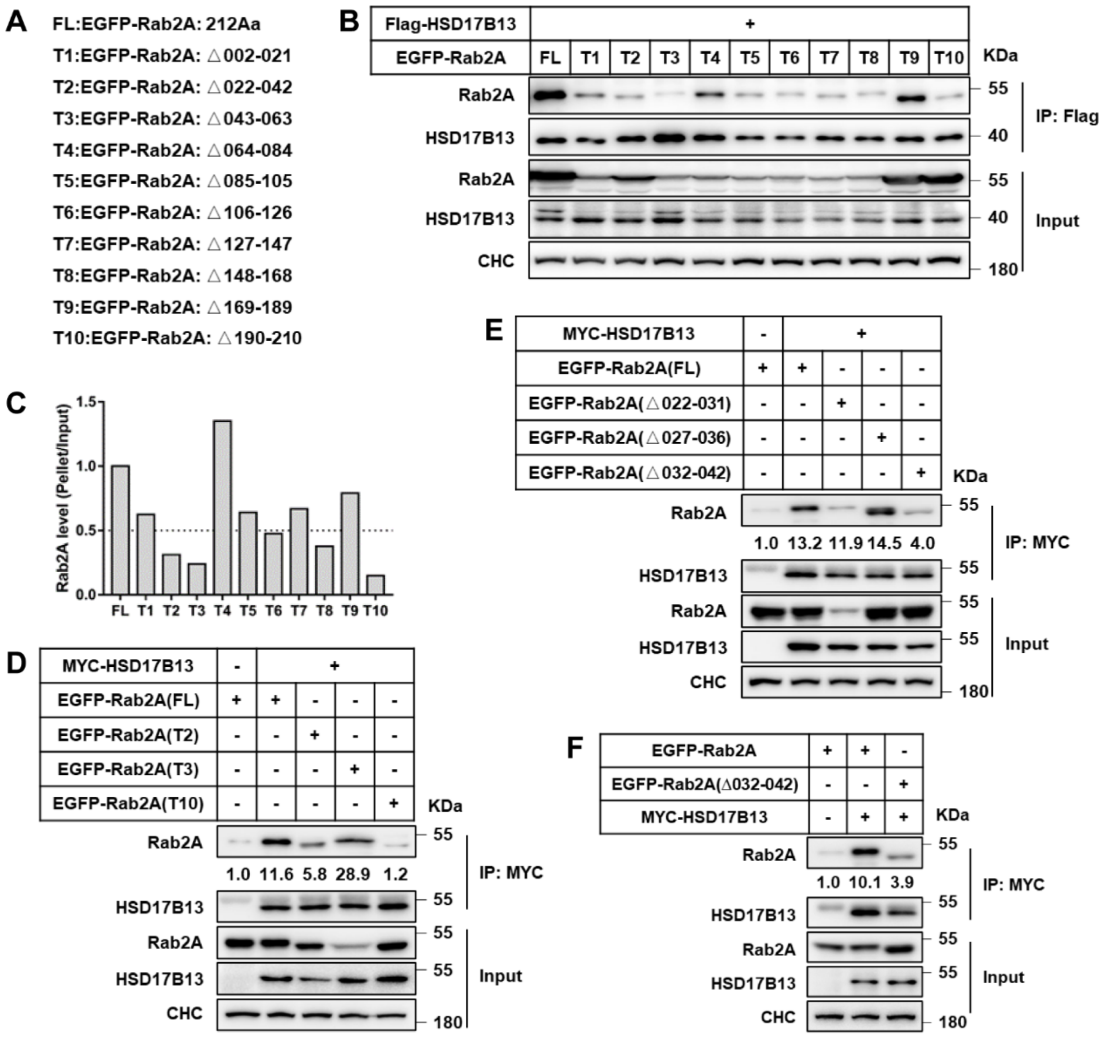

Appendix Figure S12: Fasted suppresses VLDL secretion.

(A) The impact of "Random feed" and "Fasted" states on VLDL secretion was evaluated through tyloxapol injection assay (Male, n=5 mice per group).

Data information: Data in are presented as mean ± SEM. *P* value was in determined using two-way ANOVA. * indicates $p < 0.05$.

A

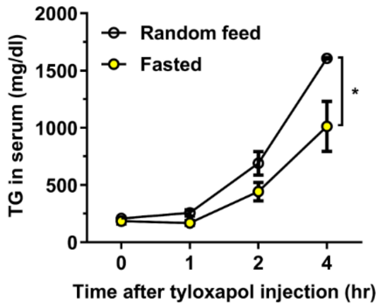

Appendix Figure S13: Quality verification of Golgi-Fractions.

(A) Golgi-Fractions were purified from the liver under "Random feed" and "Fasted" conditions via sucrose density gradient centrifugation assay. The quality of Golgi-Fractions was then validated through western blotting, utilizing Golgin-97 as Golgi marker, GRP-94 as ER marker, and GAPDH as cytosol marker.

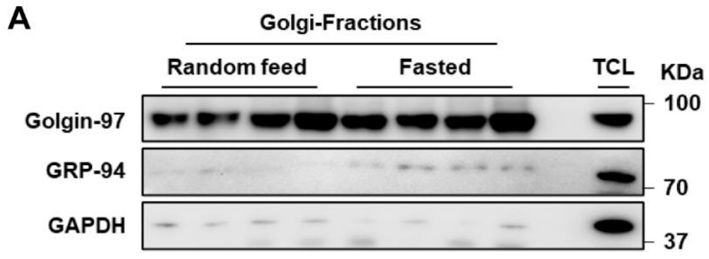

Appendix Table S1: List of 65 interacting proteins of Rab2A identified by mass spectrometry.

Abbreviatory Protein name	Peptides	Score
RAB14	2	191.01
RAB2A	8	172.24
NDUFA4	4	108.5
SLC25A10	5	97.824
HADHA	8	93.431
ACSF2	11	93.191
SEC24A	8	76.633
CYP2A12	11	70.177
DYNC1H1	10	67.79
CYP2E1	6	63.367
RAB1	2	54.944
HBB	5	53.607
UGT1A	3	51.141
RPN1	8	49.94
ACAA1	7	48.759
SUCLA2	7	48.422
DDOST	4	45.42
SDS	6	45.357
TUFM	7	44.93
AGMAT	3	44.159
ARF1	4	43.855
RPL11	3	40.082
CLTC	6	40.021
RPS12	2	39.93
HDLBP	6	38.898
SLC27A5	5	37.499
CYB5B	2	37.422
RPN2	6	37.326
GPAM	6	36.782
SLC25A15	6	36.629
ATP2A2	5	36.464
ENTPD5	4	36.17
DECR2	5	34.919
HYOU1	5	34.683
PSMC5	5	33.476
API5	5	32.719
CPT1A	5	32.236
TRAP1	5	31.847
NDUFA9	3	31.843
YTHDF3	4	31.682
RAN	5	30.965
PSMC2	5	30.732
DHDH	2	29.971
SQRDL	4	29.836
TUBB5	2	29.494
DARS	4	27.741
GNB2L1	4	26.203
MPST	4	25.838
STT3A	3	25.718
HBA	3	25.396
SEC23B	4	24.899
HACD3	3	24.892
NDUFS1	4	24.405
UQCRFS1	3	23.854
GALK1	4	23.384
EEF1G	4	22.825
HSD17B13	3	22.357
CYP2C29	3	22.205
MATR3	3	21.497
EIF3A	3	21.104
GSTZ1	3	20.32
RAB2B	3	20.215
HSD3B3	3	20.179
ECI1	3	20.096
SLC25A22	3	20.027
