# Supplementary material for: Rab2A-mediated Golgi-lipid droplet interactions support very-low-density lipoprotein secretion in hepatocytes
Source: EMBO J. 2024 Nov 4;43(24):6383–409. doi: 10.1038/s44318-024-00288-x (PMC11649929; doi:10.1038/s44318-024-00288-x)

Figure 1E

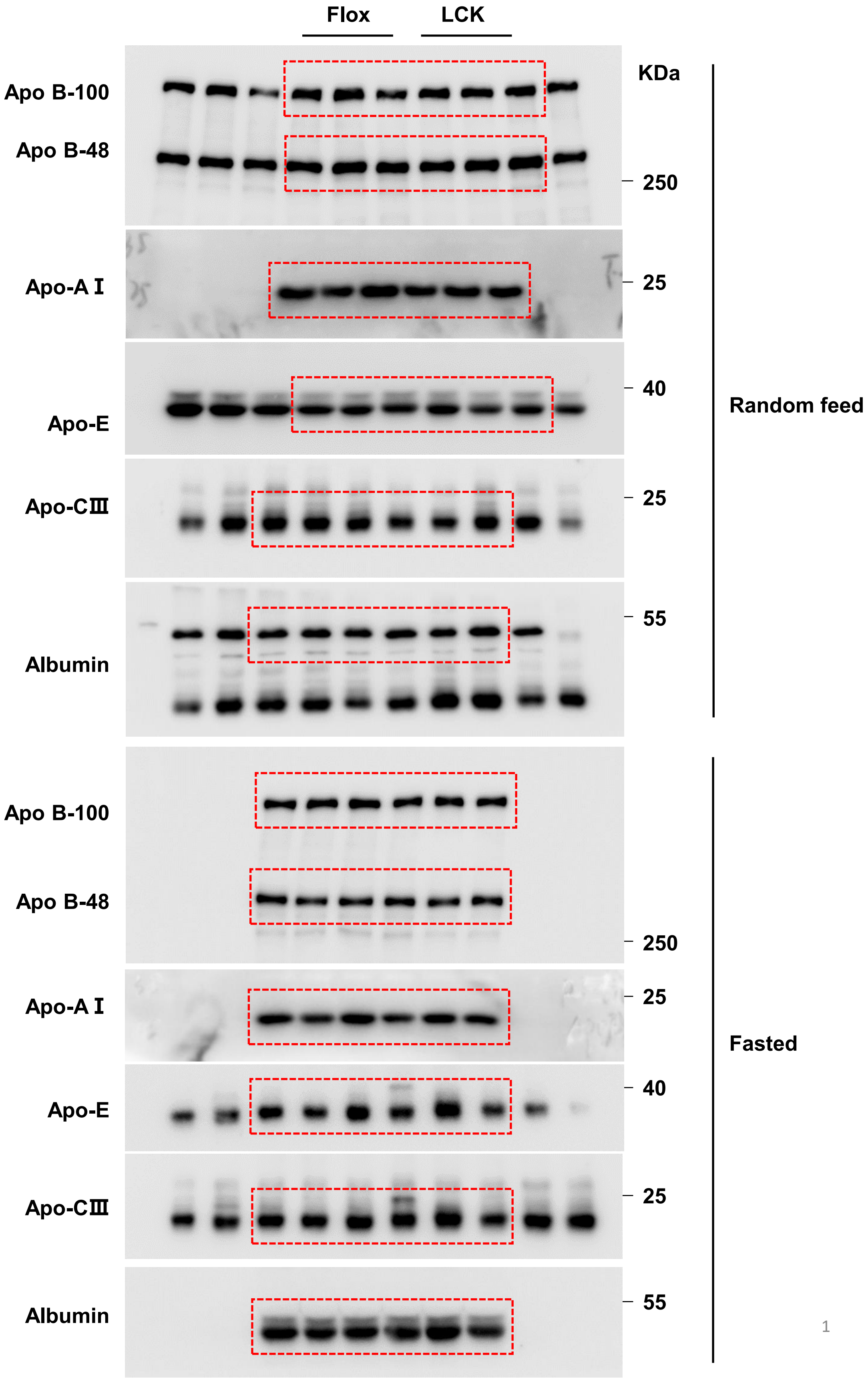
Flox
LCK
KDa
Apo B-100
Apo B-48
250
Apo-A I
25
Apo-E
40
Apo-CIII
25
Albumin
55
Random feed
Apo B-100
Apo B-48
250
Apo-A I
25
Apo-E
40
Apo-CIII
25
Albumin
55
Fasted

Figure 1K

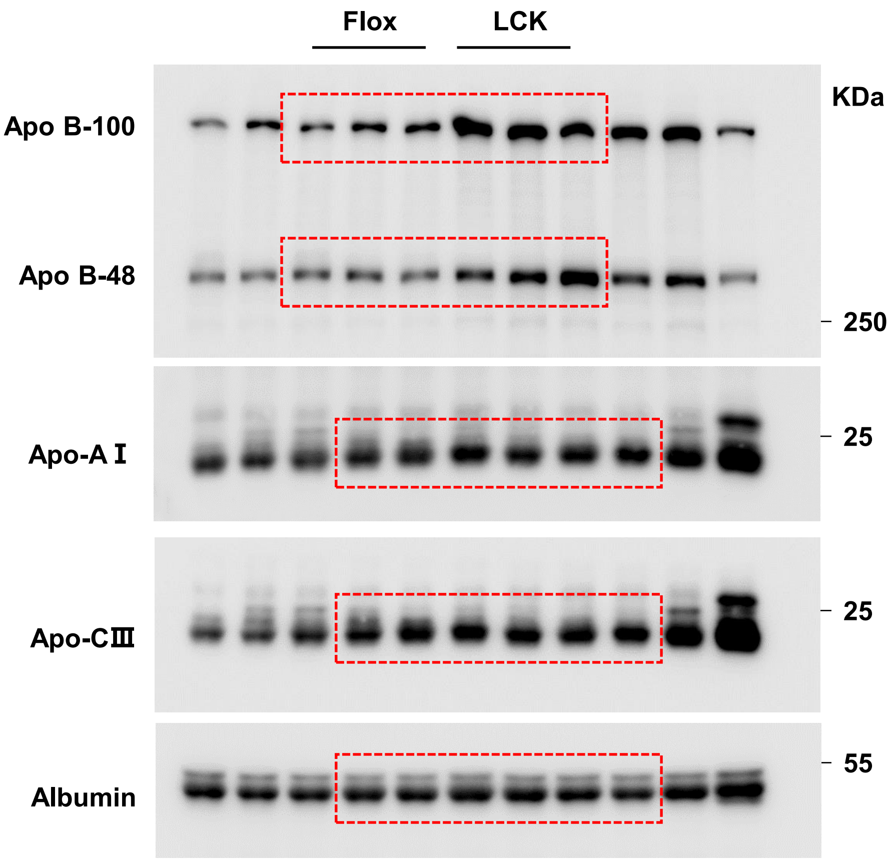

Figure 2B

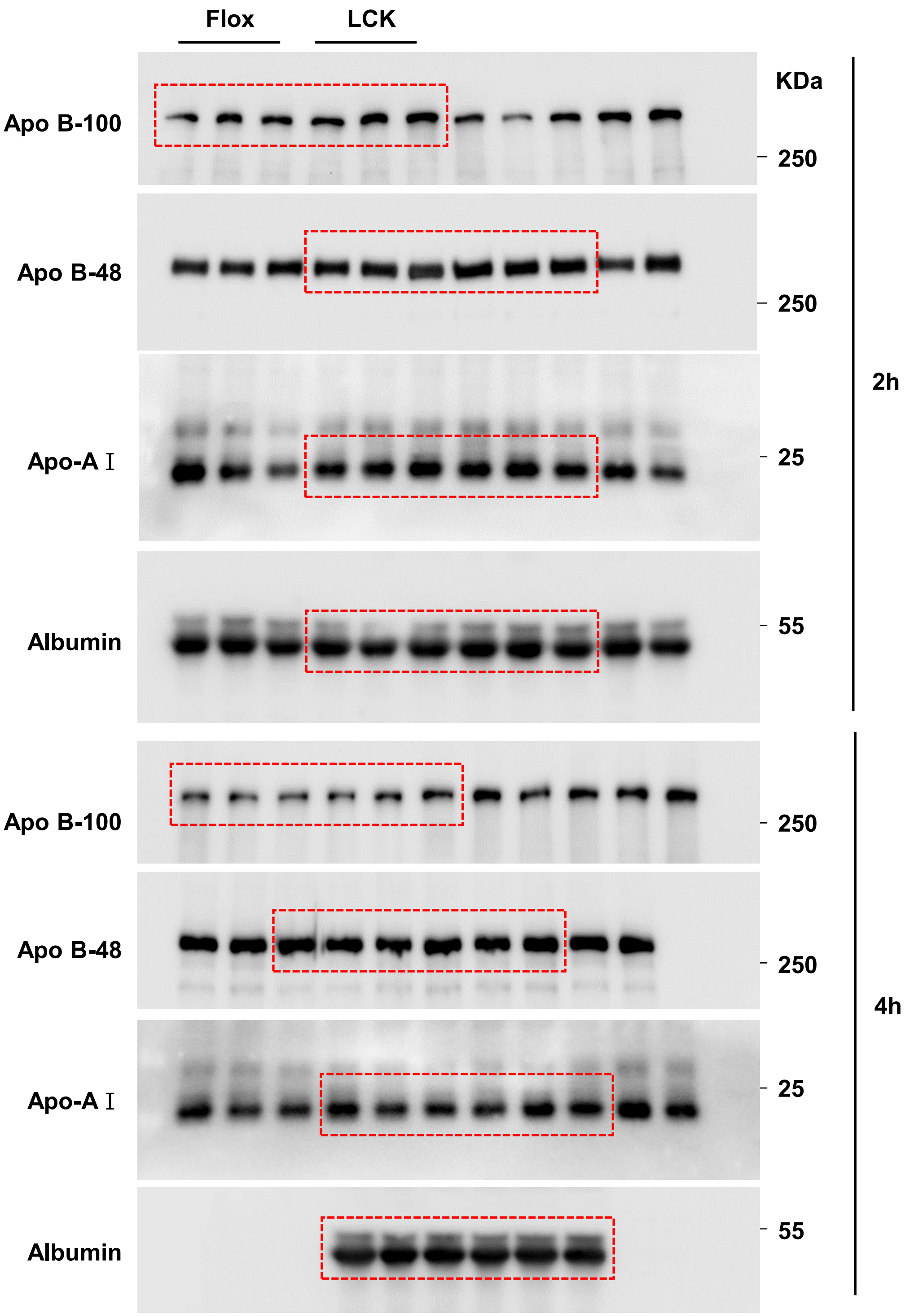
Flox
LCK
KDa
Apo B-100
250
Apo B-48
250
Apo-A Ⅰ
25
Albumin
55
2h
Apo B-100
250
Apo B-48
250
Apo-A Ⅰ
25
Albumin
55
4h

Figure 2D

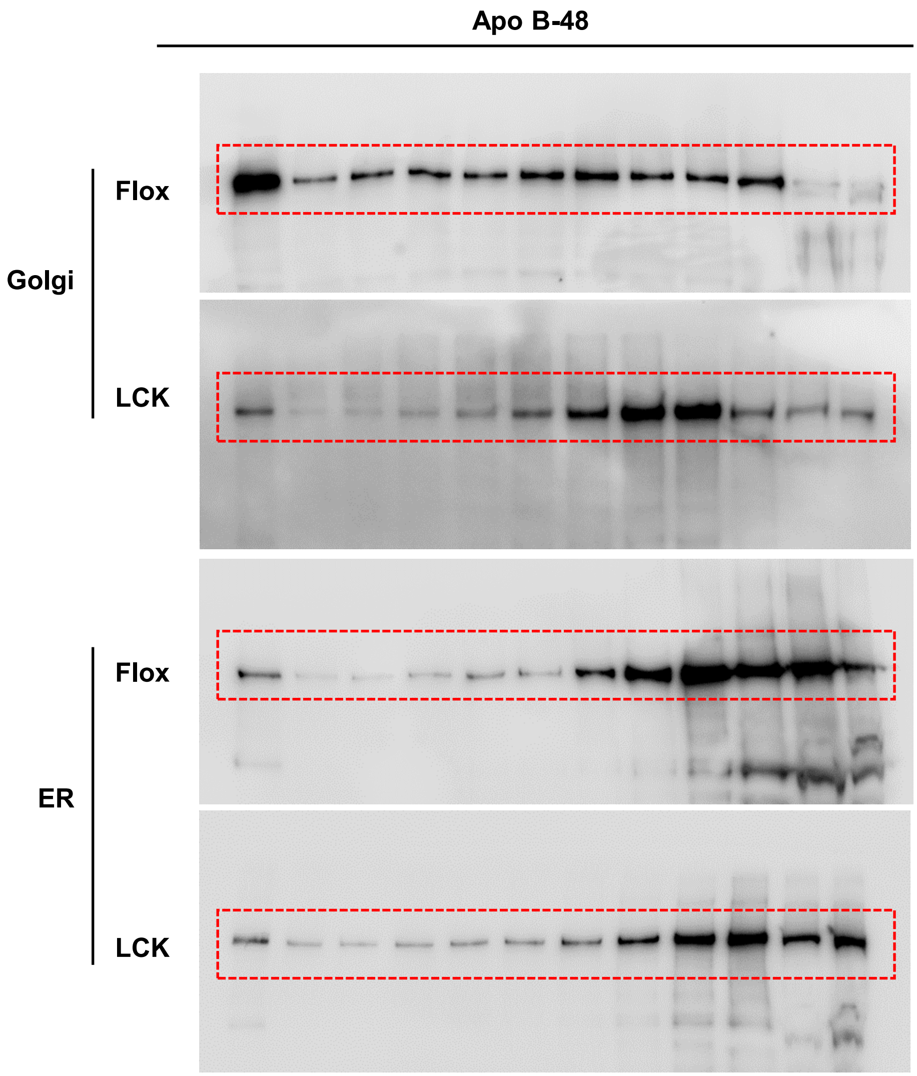

Figure 2G

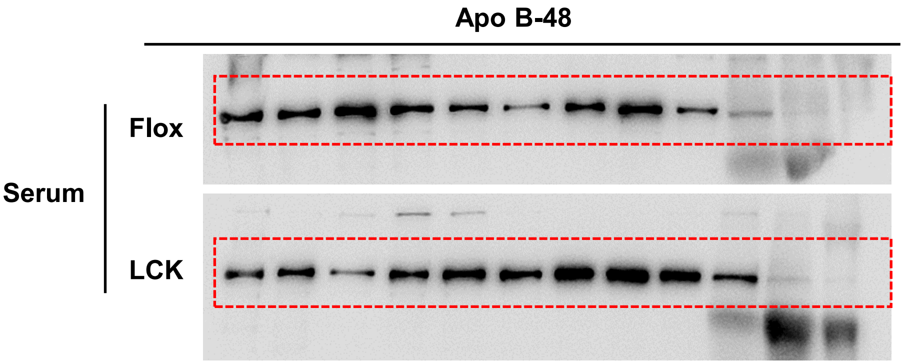

Figure 3G

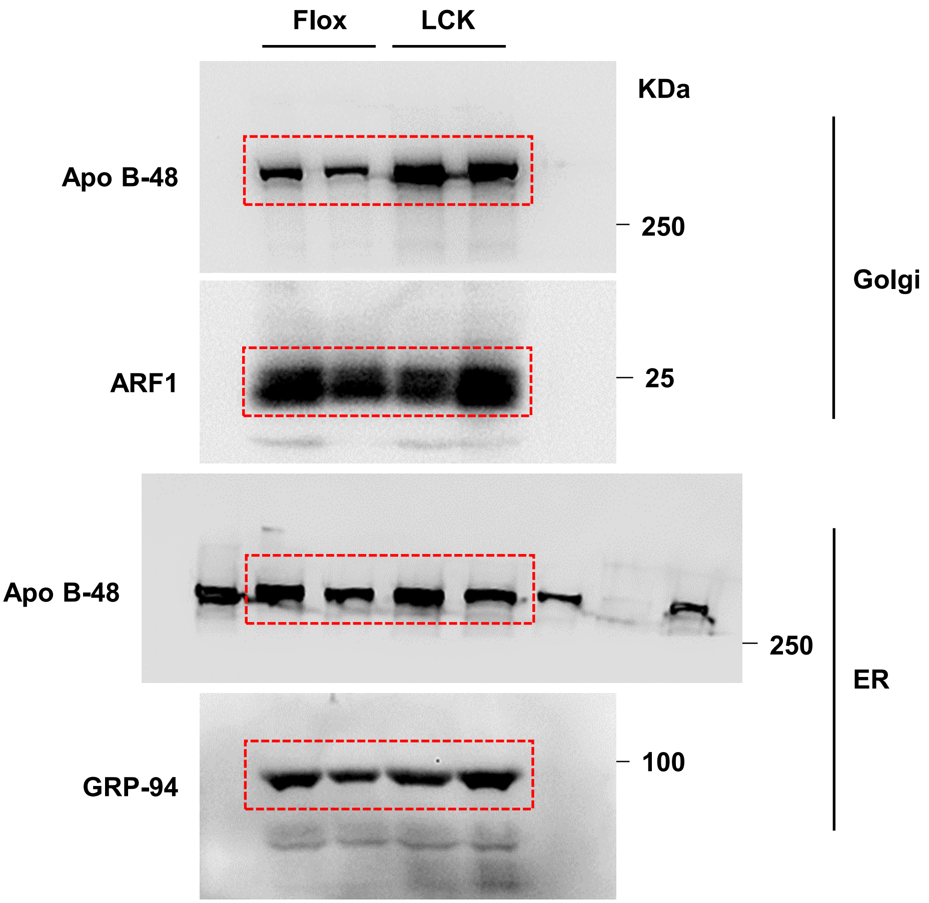

Figure 3J

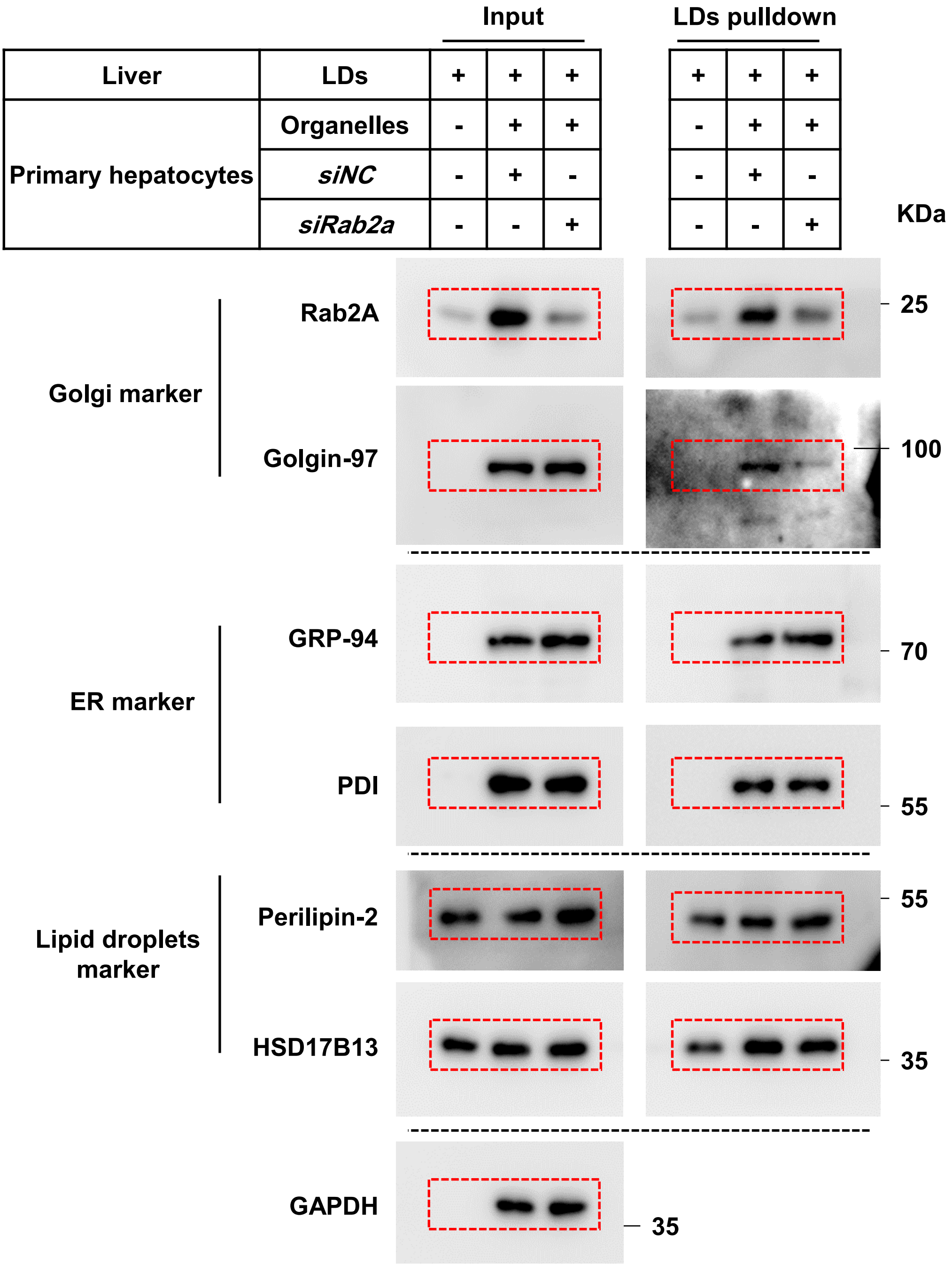
Input
LDs pulldown
Liver
LDs
Organelles
Primary hepatocytes
siNC
siRab2a
KDa
Golgi marker
Rab2A
Golgin-97
ER marker
GRP-94
PDI
Lipid droplets marker
Perilipin-2
HSD17B13
GAPDH
25
100
70
55
55
35
35

Figure 4A

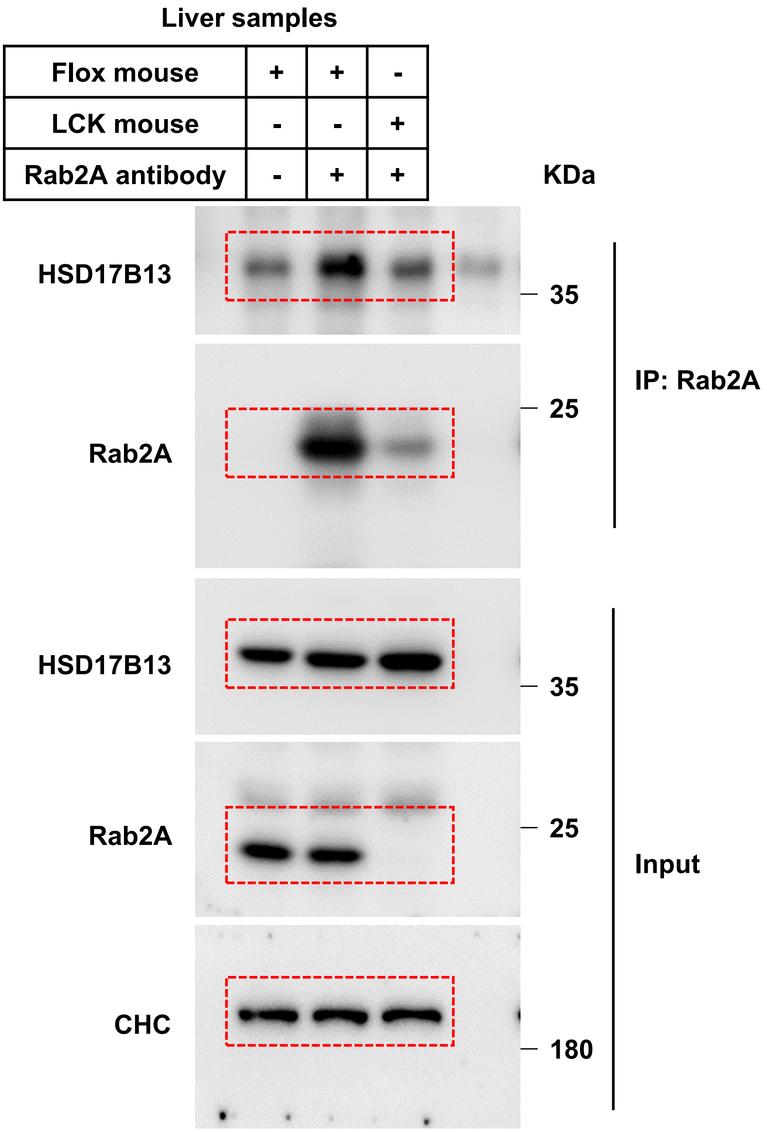
Liver samples
Flox mouse + + -
LCK mouse - - +
Rab2A antibody - + +
KDa
HSD17B13
35
Rab2A
25
IP: Rab2A
HSD17B13
35
Rab2A
25
Input
CHC
180

Figure 4D

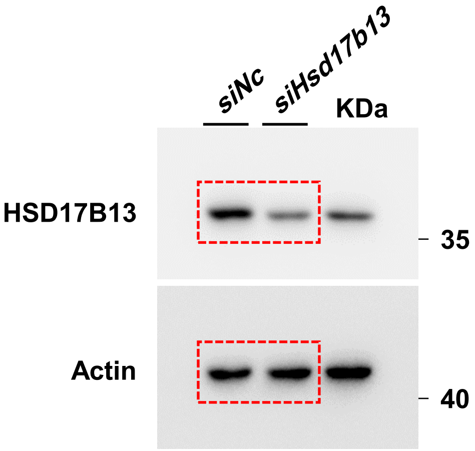

Figure 4H

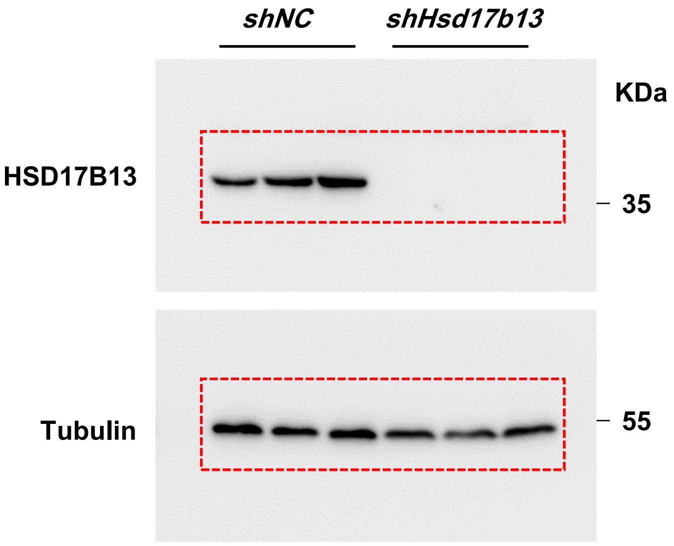

Figure 4I

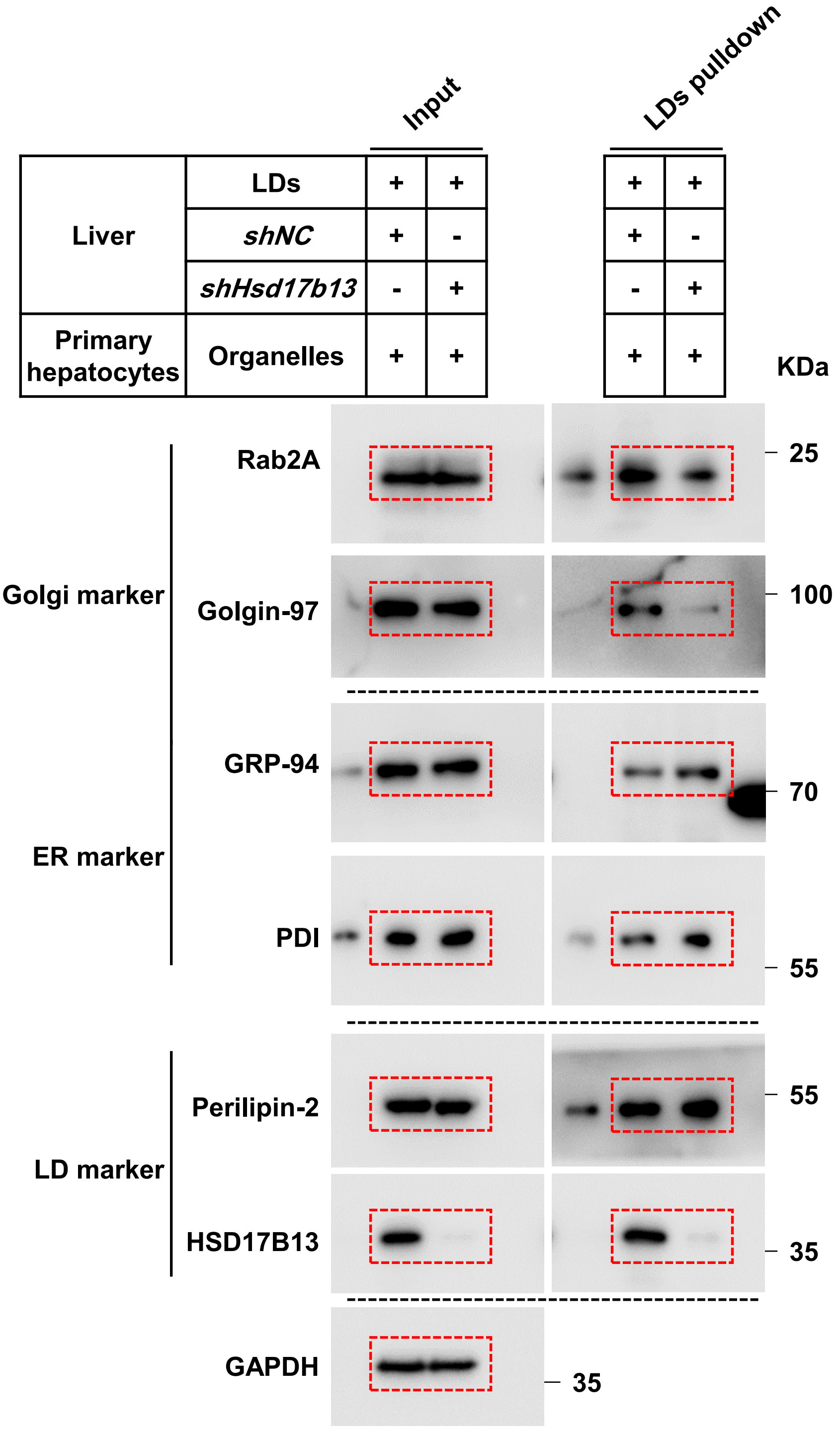
Input
LDs pulldown
Liver
LDs
shNC
shHsd17b13
Primary hepatocytes
Organelles
KDa
Golgi marker
Rab2A
Golgin-97
ER marker
GRP-94
PDI
LD marker
Perilipin-2
HSD17B13
GAPDH
25
100
70
55
55
35
35

Figure 5A

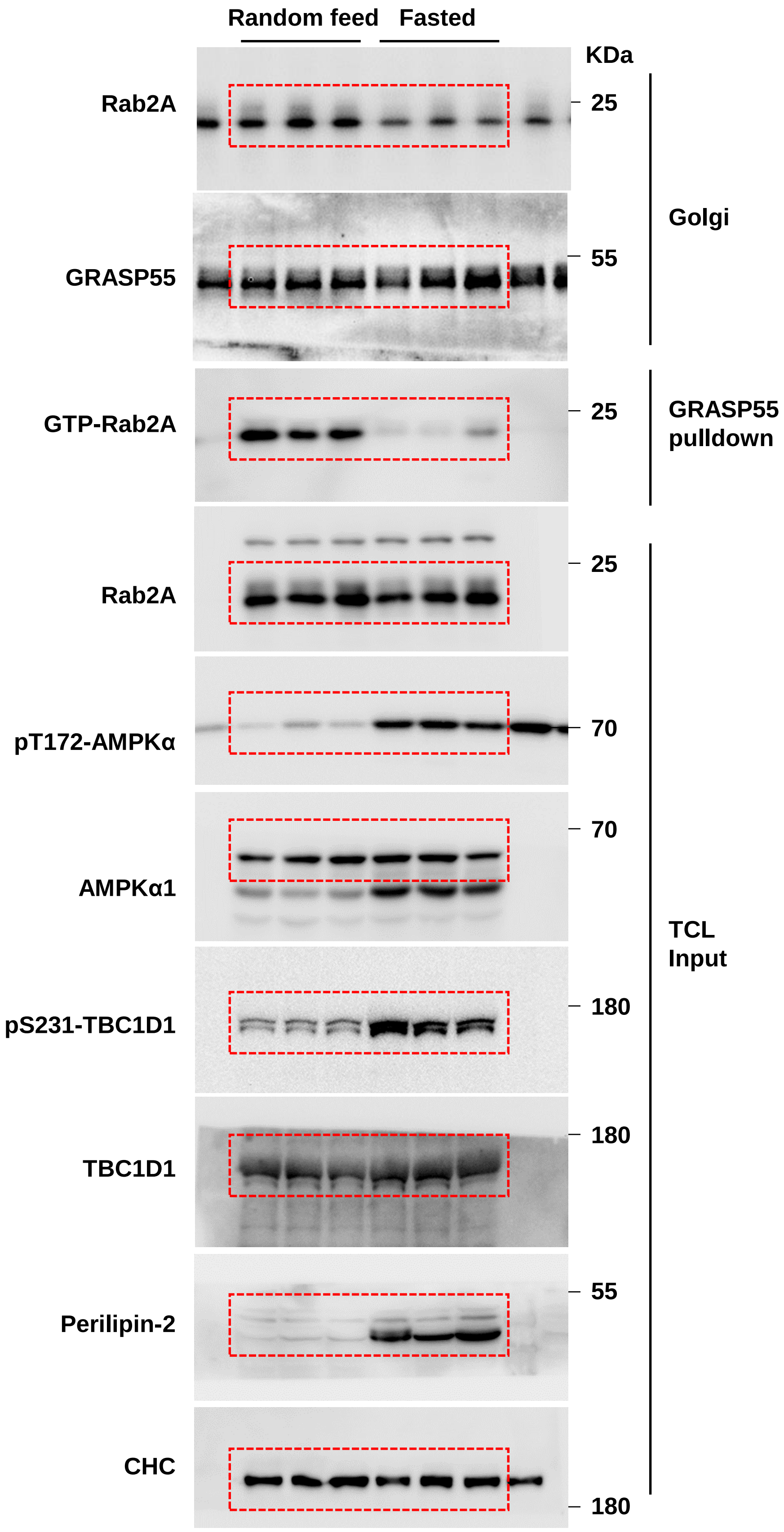

Random feed
Fasted
KDa
Rab2A
25
GRASP55
55
Golgi
GTP-Rab2A
25
GRASP55
pulldown
Rab2A
25
pT172-AMPKα
70
AMPKα1
70
pS231-TBC1D1
180
TBC1D1
180
Perilipin-2
55
CHC
180
TCL
Input

Figure 5C

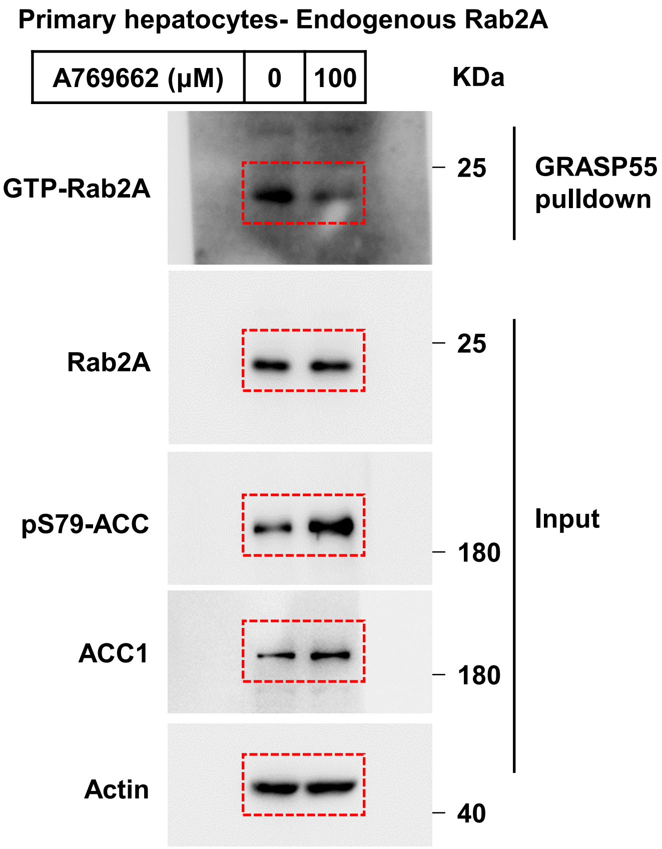
Primary hepatocytes- Endogenous Rab2A
A769662 (μM)
0
100
KDa
GTP-Rab2A
25
GRASP55
pulldown
Rab2A
25
pS79-ACC
180
Input
ACC1
180
Actin
40

Figure 5F

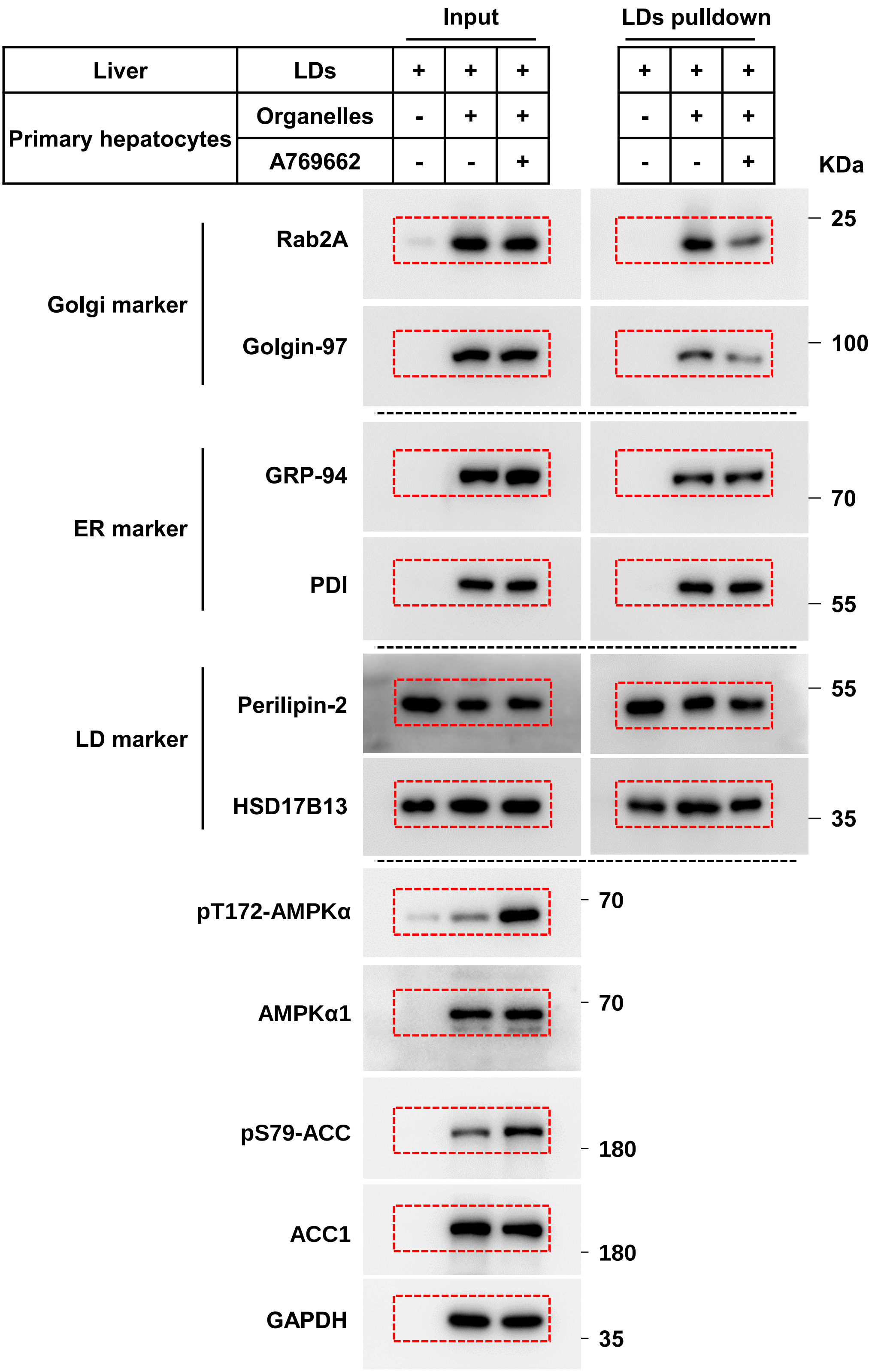

Input
LDs pulldown
Liver
LDs
Primary hepatocytes
Organelles
A769662
KDa
Golgi marker
Rab2A
Golgin-97
ER marker
GRP-94
PDI
LD marker
Perilipin-2
HSD17B13
pT172-AMPKα
AMPKα1
pS79-ACC
ACC1
GAPDH
25
100
70
55
55
35
70
70
180
180
35

Figure 5G

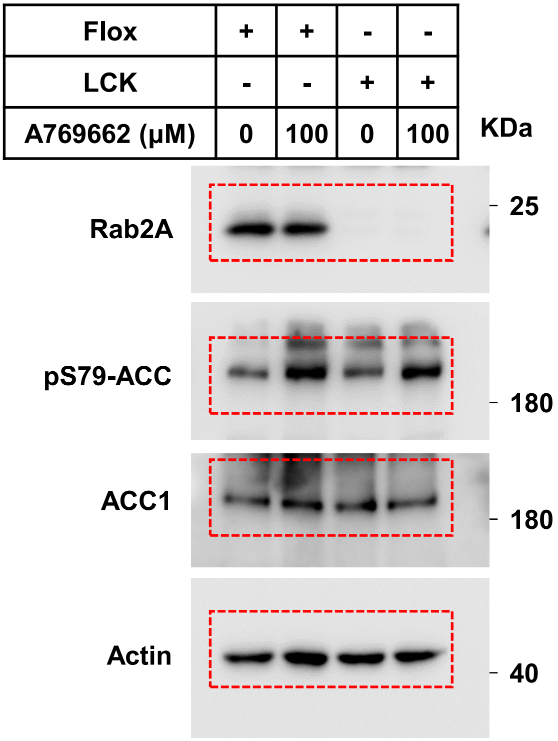

Flox
+
+
-
-
LCK
-
-
+
+
A769662 (μM)
0
100
0
100
KDa
Rab2A
25
pS79-ACC
180
ACC1
180
Actin
40

Figure EV3A

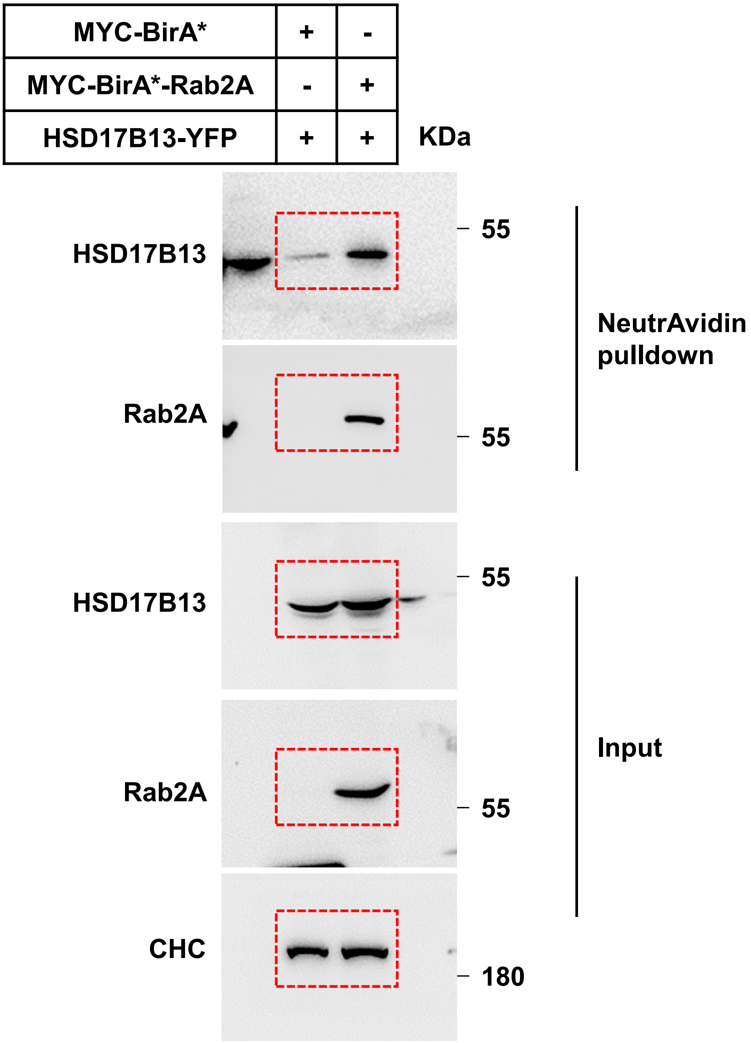
MYC-BirA*
MYC-BirA*-Rab2A
HSD17B13-YFP
+
-
+
KDa
HSD17B13
Rab2A
HSD17B13
Rab2A
CHC
55
55
55
55
180
NeutrAvidin pulldown
Input

Figure EV3B

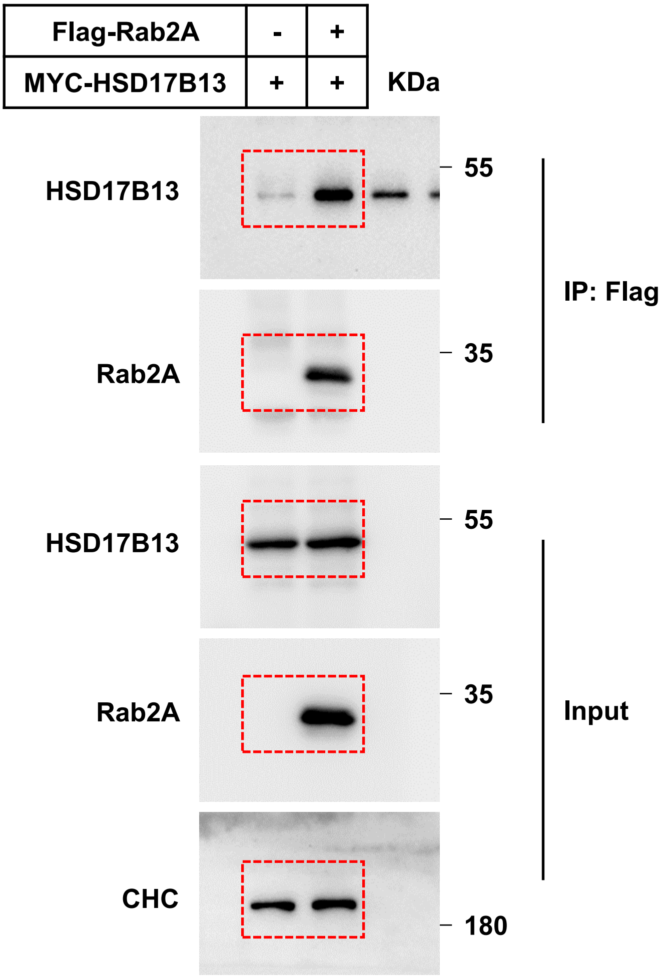

Flag-Rab2A - +
MYC-HSD17B13 + +
KDa
HSD17B13
55
Rab2A
35
IP: Flag
HSD17B13
55
Rab2A
35
Input
CHC
180

Figure EV3C

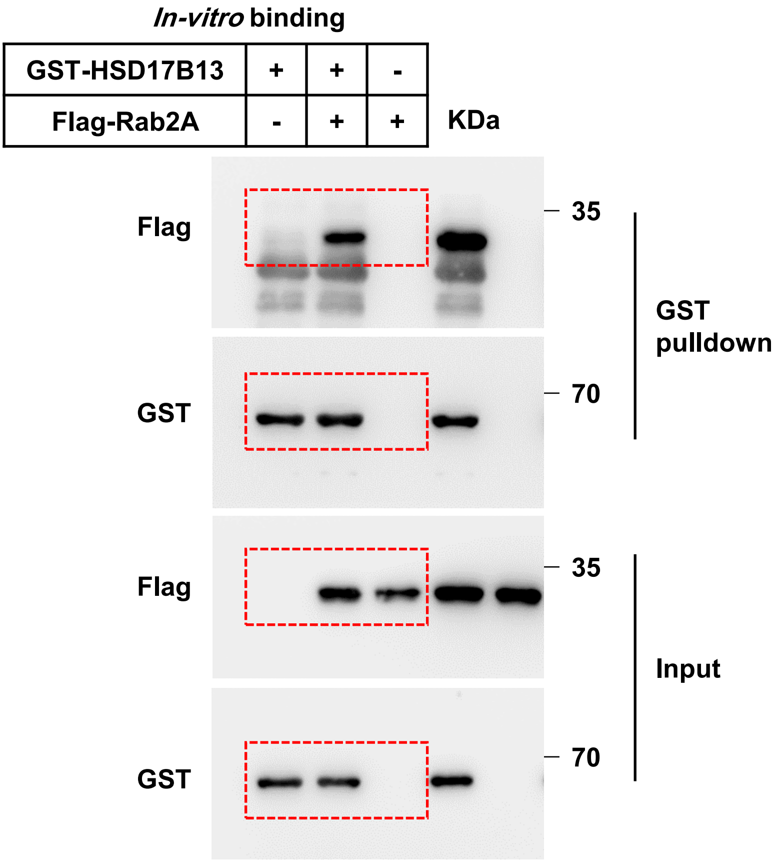

In-vitro binding
GST-HSD17B13 + + -
Flag-Rab2A - + +
KDa
Flag
GST
Flag
GST
35
70
35
70
GST pulldown
Input

Figure EV3F

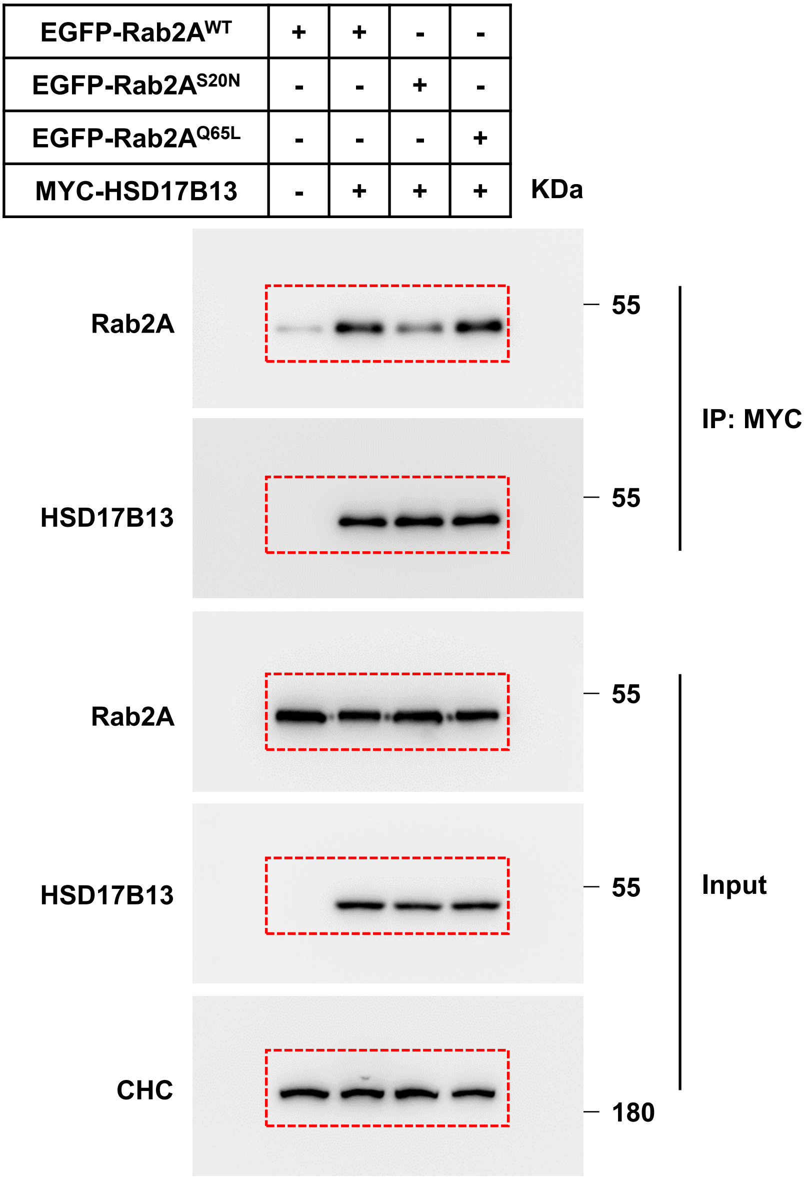

EGFP-Rab2A^{WT} + + - -
EGFP-Rab2A^{S20N} - - + -
EGFP-Rab2A^{Q65L} - - - +
MYC-HSD17B13 - + + +
KDa
Rab2A
HSD17B13
Rab2A
HSD17B13
CHC
55
55
55
55
180
IP: MYC
Input

Appendix Figure S8A

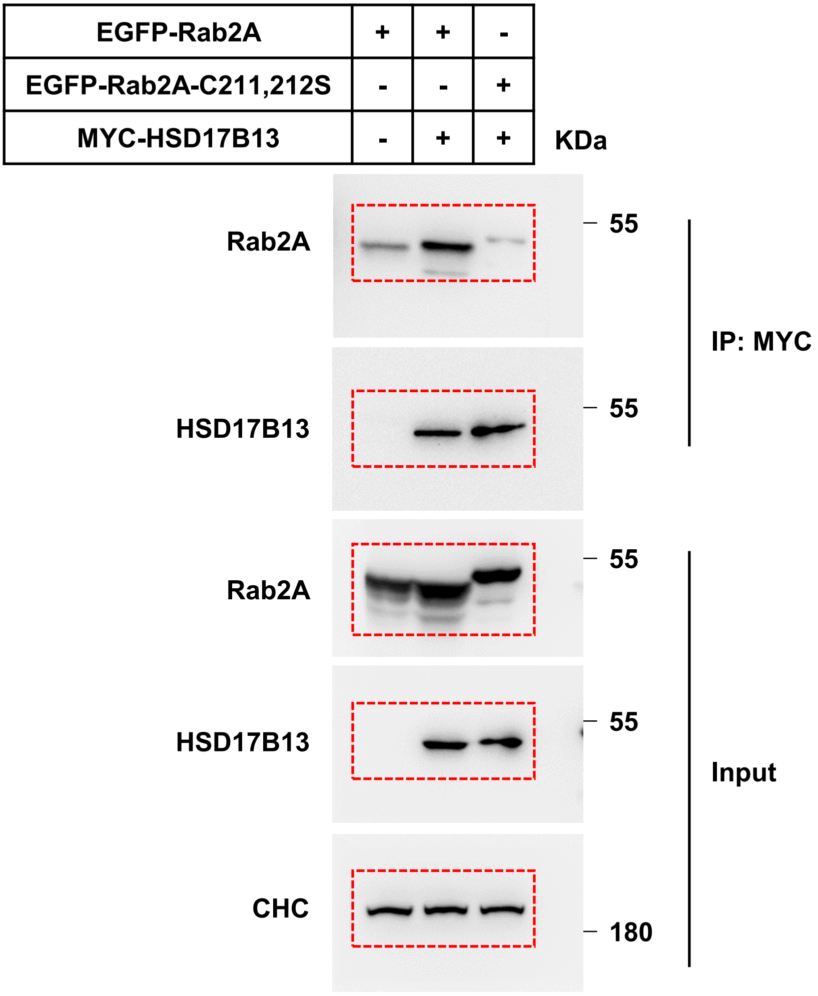

Appendix Figure S8C

EGFP-Rab2A	+	+	-
EGFP-Rab2A(Δ190-210)	-	-	+
MYC-HSD17B13	-	+	+

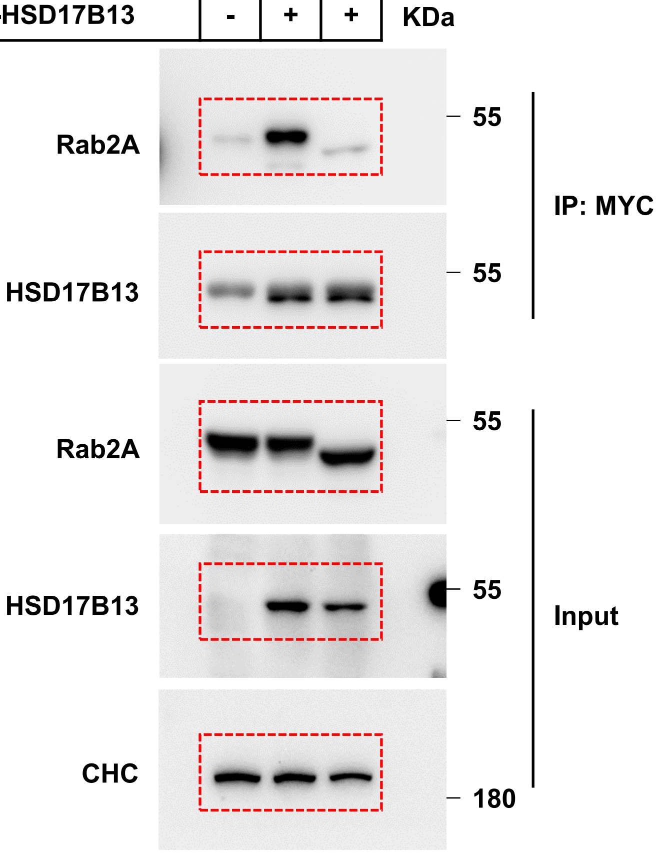

Appendix Figure S8E

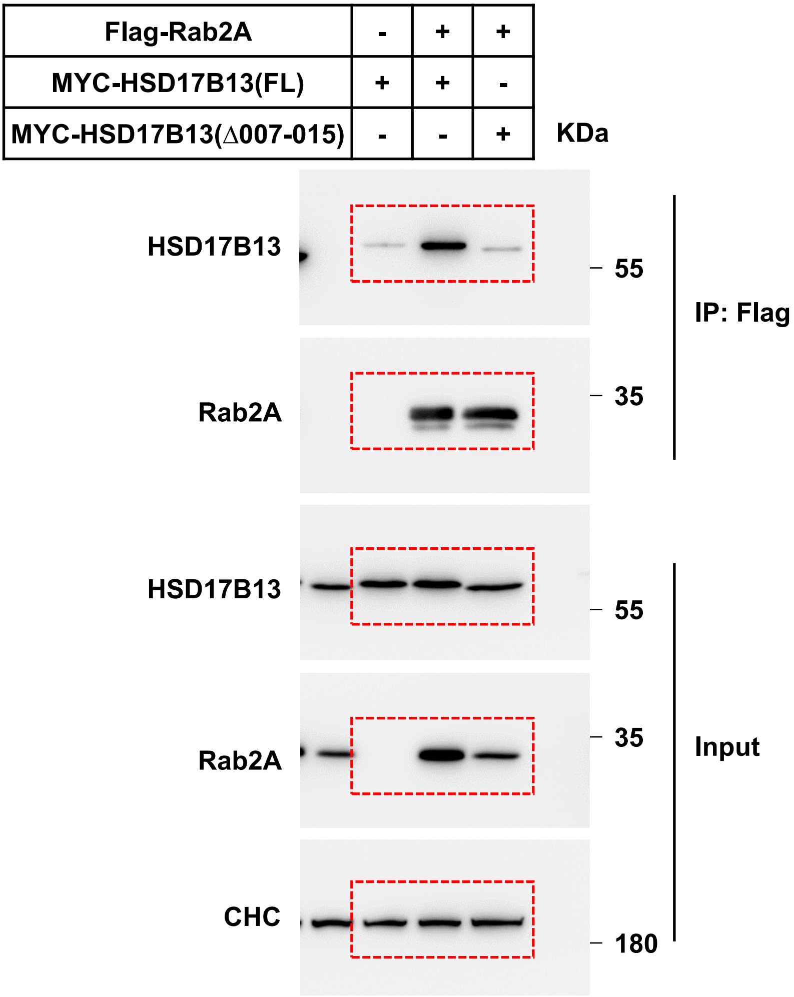

Supplement: Supplementary file 9 — Figure Source Data - Uncropped gel images [file 44318_2024_288_MOESM9_ESM.zip › Source_data-Uncropped_gel_images.pdf]
