# Supplementary material for: Rab2A-mediated Golgi-lipid droplet interactions support very-low-density lipoprotein secretion in hepatocytes
Source: EMBO J. 2024 Nov 4;43(24):6383–409. doi: 10.1038/s44318-024-00288-x (PMC11649929; doi:10.1038/s44318-024-00288-x)
Supplement: Supplementary file 10 — Expanded View Figures [file 44318_2024_288_MOESM10_ESM.pdf]

Expanded View Figures

Figure EV1. Evaluating the rates of lipid secretion in Flox and LCK mice.

(**A**) Triglyceride (TG) secretion in female mice were assessed through a tyloxapol injection assay ($n = 7$ vs. 6 mice) ($P = 0.0021$). (**B**) A visual examination of blood transparency in Flox and LCK mice was conducted four hours post-tyloxapol injection. (**C**, **D**) The evaluation of the TG secretion in primary hepatocytes involved quantifying the secretion level of TG (**C**) ($P < 0.0001$) and Apo B-48 (**D**), with normalization of Flox samples' secretion level at 1-hour to 1. (**E–H**) The schematic representation outlines the timeline for the *Apob* knockdown assay (**E**), followed by the validation of Apo B-48 proteins levels (**F**) and assessment of serum TG (**G**) (Flox (*shNC* vs. *shApob*), $P < 0.0001$; *shNC* (Flox vs. LCK), $P < 0.0001$; LCK (*shNC* vs. *shApob*), $P = 0.0079$) and TC (**H**) (Flox (*shNC* vs. *shApob*), $P < 0.0001$; *shNC* (Flox vs. LCK), $p = 0.0009$; LCK (*shNC* vs. *shApob*), $P = 0.0034$) levels under "Fasted" condition (Male, $n = 10$ vs. 5 vs. 7 vs. 8 mice). Data information: Data in (**A**, **C**, **G**, **H**) are presented as mean ± SEM. Circles in (**G**, **H**) correspond to individual mice. *P* values in (**A**, **C**) were determined using two-way ANOVA. *P* values in (**G**, **H**) were determined using unpaired two-tailed Student's *t*-test. n.s. indicates no significant difference ($P > 0.05$), ** indicates $P < 0.01$; *** indicates $P < 0.001$.

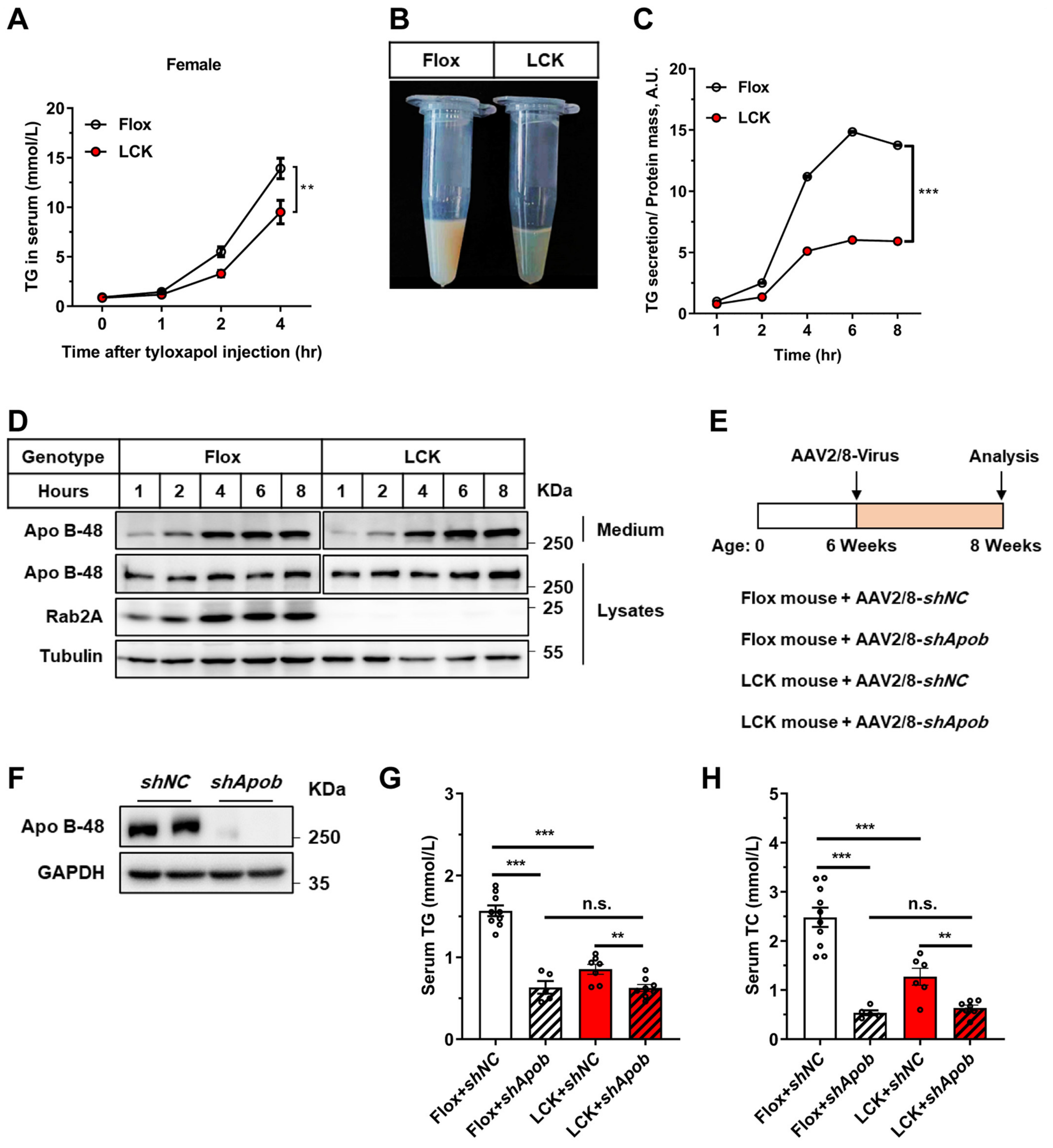

A
Female
TG in serum (mmol/L)
Flox
LCK
**
Time after tyloxapol injection (hr)
B
Flox
LCK
C
TG secretion/ Protein mass, A.U.
Flox
LCK
***
Time (hr)
D
Genotype
Flox
LCK
Hours
1 2 4 6 8 1 2 4 6 8
KDa
Apo B-48
250
Medium
Apo B-48
250
Rab2A
25
Lysates
Tubulin
55
E
AAV2/8-Virus
Analysis
Age: 0
6 Weeks
8 Weeks
Flox mouse + AAV2/8-shNC
Flox mouse + AAV2/8-shApob
LCK mouse + AAV2/8-shNC
LCK mouse + AAV2/8-shApob
F
shNC
shApob
KDa
Apo B-48
250
GAPDH
35
G
Serum TG (mmol/L)
***
***
n.s.
**
Flox+shNC
Flox+shApob
LCK+shNC
LCK+shApob
H
Serum TC (mmol/L)
***
***
n.s.
**
Flox+shNC
Flox+shApob
LCK+shNC
LCK+shApob

© The Author(s)

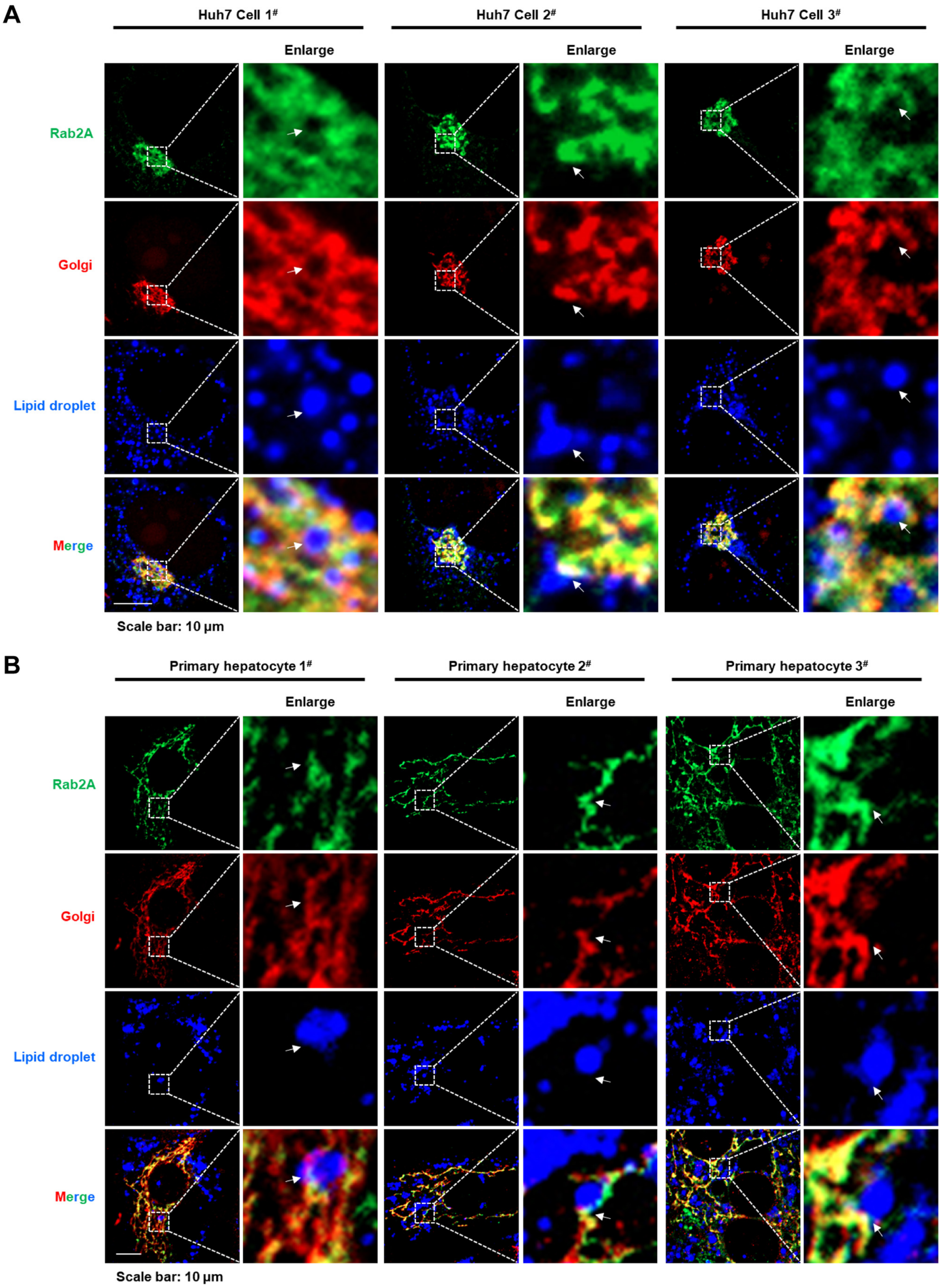
A
Huh7 Cell 1#
Huh7 Cell 2#
Huh7 Cell 3#
Enlarge
Rab2A
Golgi
Lipid droplet
Merge
Scale bar: 10 μm
B
Primary hepatocyte 1#
Primary hepatocyte 2#
Primary hepatocyte 3#
Enlarge
Rab2A
Golgi
Lipid droplet
Merge
Scale bar: 10 μm

◀ **Figure EV2. Golgi-localized Ras-related protein Rab-2A (Rab2A) contacts with LDs.**

(**A**, **B**) Immunofluorescence analysis elucidated Rab2A-mediated organelle contacts between Golgi and LDs in Huh7 cells (**A**) and primary hepatocytes (**B**) with overexpression of EGFP-Rab2A plasmids. Golgi was labeled using a primary antibody targeting GM130, while LDs were stained with Bodipy (The white arrow indicates different types of contact points).

 © The Author(s)

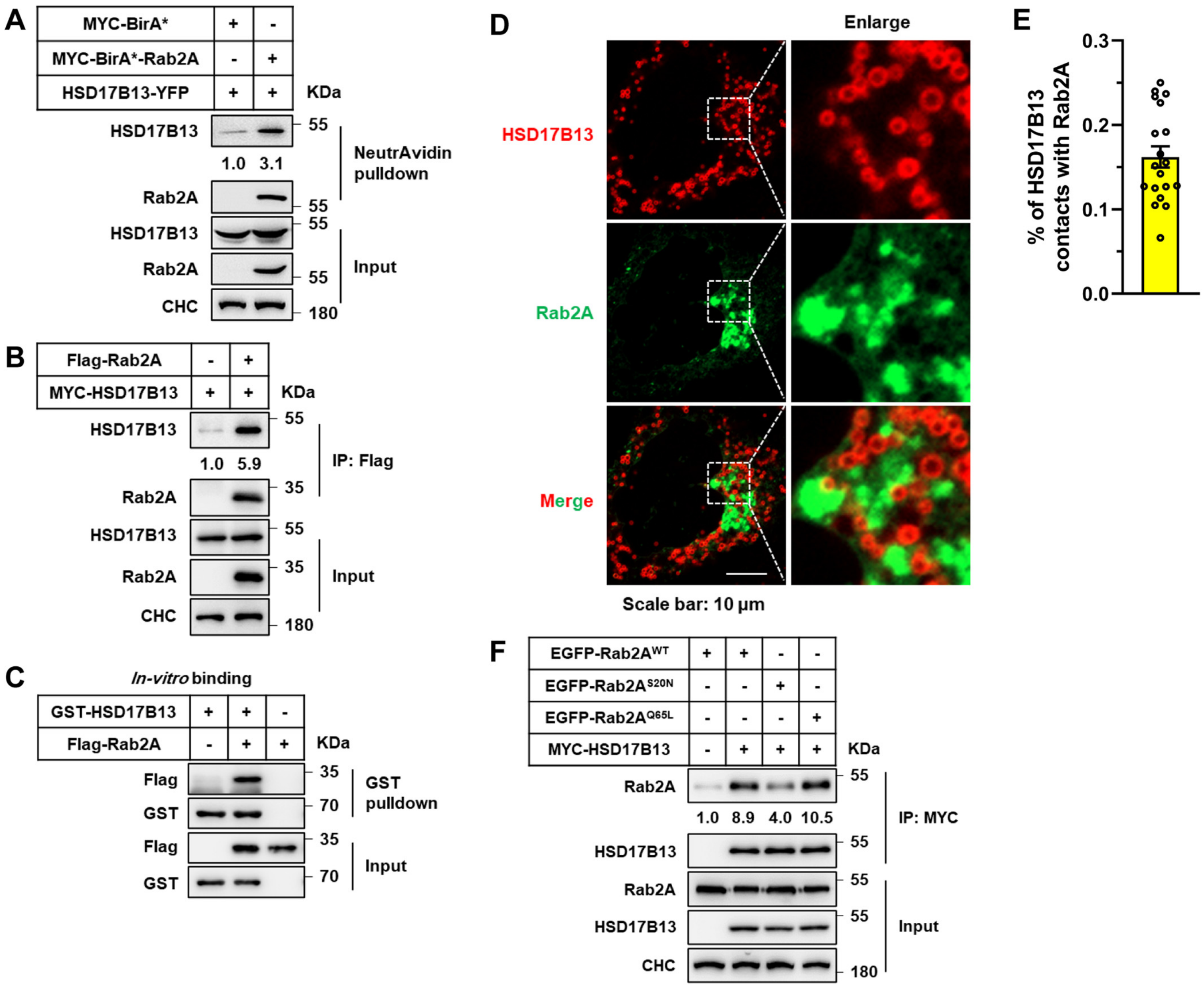

Figure EV3. Confirming the binding efficiency between Rab2A and 17-beta-hydroxysteroid dehydrogenase 13 (HSD17B13) using exogenously expressed Rab2A and HSD17B13.

(**A**) HEK293T cells were transfected with the BirA*-Rab2A and HSD17B13 plasmids. Rab2A-HSD17B13 binding was evaluated via NeutrAvidin beads pulldown, followed by visualization using Western blotting. (**B**) HEK293T cells were transfected with Flag-Rab2A and MYC-HSD17B13 plasmids, followed by a Flag-affinity beads pulldown assay to confirm Rab2A and HSD17B13 binding. (**C**) In vitro binding assay was conducted using purified GST-HSD17B13 and Flag-Rab2A proteins. (**D**, **E**) Huh7 cells underwent transfection with HSD17B13-mCherry and EGFP-Rab2A plasmids. Immunofluorescence was employed to observe the binding efficiency between Rab2A and HSD17B13. Representative images are shown (**D**), and the percentage of HSD17B13 contacting with Rab2A was quantified (**E**) ($n = 19$ cells). (**F**) The regulatory effect of Rab2A activity on the binding between Rab2A and HSD17B13 was examined in HEK293T cells by transfecting with different types of Rab2A plasmids, followed by MYC-affinity beads pulldown assay. Data information: Data in (**E**) are presented as mean ± SEM. Circles in (**E**) correspond to individual Huh7 cell. Source data are available online for this figure.

© The Author(s)

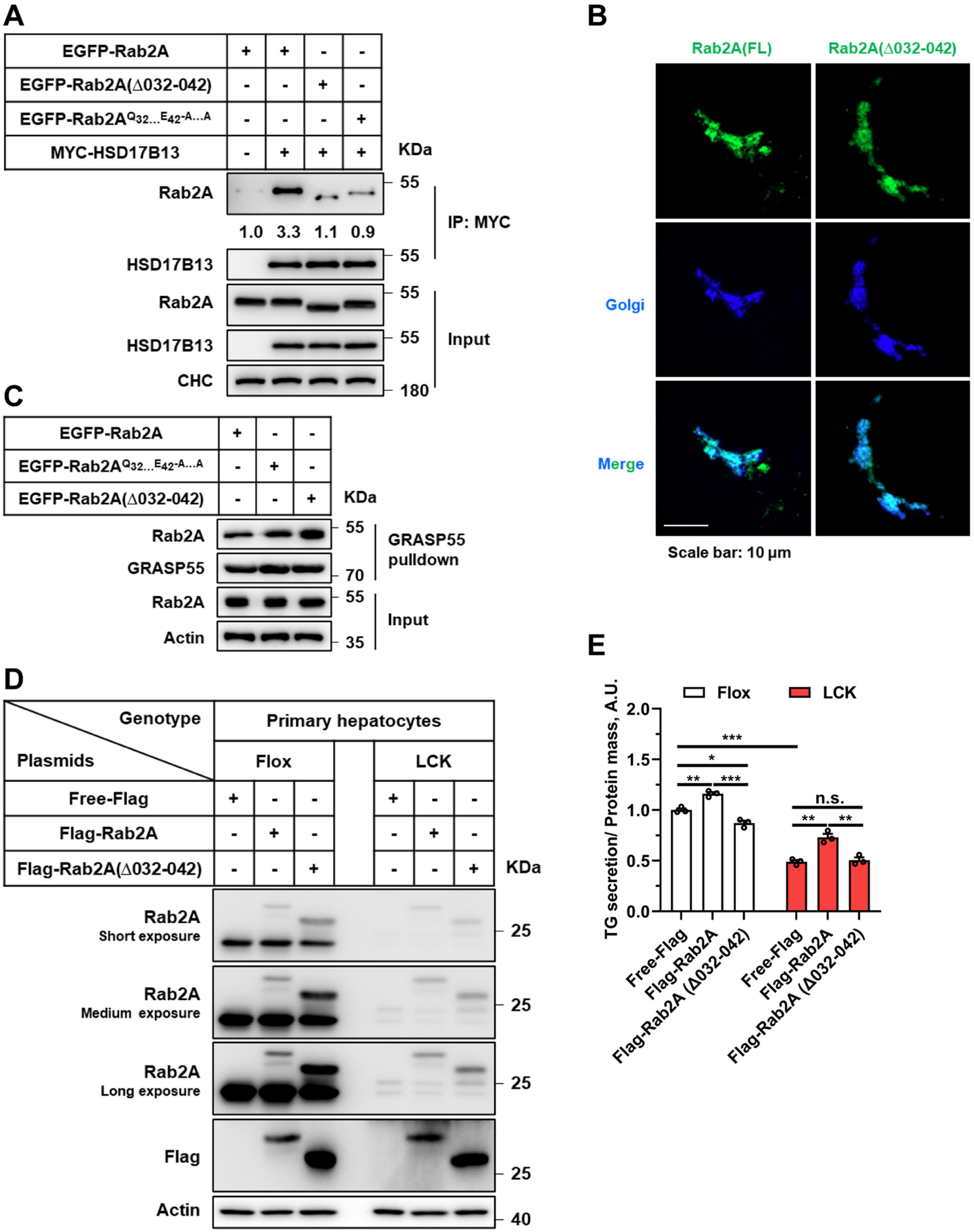
A
EGFP-Rab2A
EGFP-Rab2A(Δ032-042)
EGFP-Rab2A$^{Q32...E42-A...A}$
MYC-HSD17B13
KDa
Rab2A
1.0 3.3 1.1 0.9
HSD17B13
IP: MYC
Rab2A
HSD17B13
CHC
Input
55
180
B
Rab2A(FL)
Rab2A(Δ032-042)
Golgi
Merge
Scale bar: 10 μm
C
EGFP-Rab2A
EGFP-Rab2A$^{Q32...E42-A...A}$
EGFP-Rab2A(Δ032-042)
KDa
Rab2A
GRASP55
GRASP55 pulldown
Rab2A
Actin
Input
55
70
35
D
Genotype
Plasmids
Primary hepatocytes
Flox
LCK
Free-Flag
Flag-Rab2A
Flag-Rab2A(Δ032-042)
KDa
Rab2A Short exposure
Rab2A Medium exposure
Rab2A Long exposure
Flag
Actin
25
40
E
Flox
LCK
TG secretion/ Protein mass, A.U.
0.0
0.5
1.0
1.5
2.0
***
*
**
***
n.s.
Free-Flag
Flag-Rab2A
Flag-Rab2A (Δ032-042)

 © The Author(s)

Figure EV4. Blocking the binding between Rab2A and HSD17B13 using a Rab2A mutant protein suppresses very-low-density lipoprotein (VLDL) secretion in primary hepatocytes.

(**A–E**) Examination of the effects of Rab2A and HSD17B13 binding on TG secretion involving multiple steps. First, vital amino acids for complex binding in Rab2A were identified via Western blotting in HEK293T cells after transfection with relative plasmids (**A**). Second, the localization of mutant Rab2A protein was assessed using immunofluorescence in Huh7 cells following transfection with EGFP-Rab2A plasmids, with Golgi labeling accomplished using RCAS1-BFP (**B**). Third, the activity of both wild-type and mutant Rab2A proteins was detected using a GST-GRASP55 pulldown assay to assess influence of specific mutations on Rab2A's activity (**C**). Finally, the impact of deficient complex binding on TG secretion was evaluated in Flox and LCK primary hepatocytes after protein overexpression with the AAV-virus system (**D**). The medium was collected for testing, with values in Flox samples with Free-Flag overexpression normalized to 1 (**E**) (Flox hepatocytes (Free-Flag vs. Flag-Rab2A), $P = 0.0025$; Flox hepatocytes (Free-Flag vs. Flag-Rab2A (Δ032-042)), $P = 0.0101$; Flox hepatocytes (Flag-Rab2A vs. Flag-Rab2A (Δ032-042)), $P = 0.0005$; Free-Flag (Flox vs. LCK), $P < 0.0001$; LCK hepatocytes (Free-Flag vs. Flag-Rab2A), $P = 0.0030$; LCK hepatocytes (Flag-Rab2A vs. Flag-Rab2A (Δ032-042)), $P = 0.0063$). Data information: Data in (**E**) are presented as mean ± SEM. Circles in (**E**) correspond to individual test. *P* values in (**E**) were determined using unpaired two-tailed Student's *t*-test. n.s. indicates no significant difference ($P > 0.05$), * indicates $P < 0.05$; ** indicates $P < 0.01$; *** indicates $P < 0.001$.

© The Author(s)

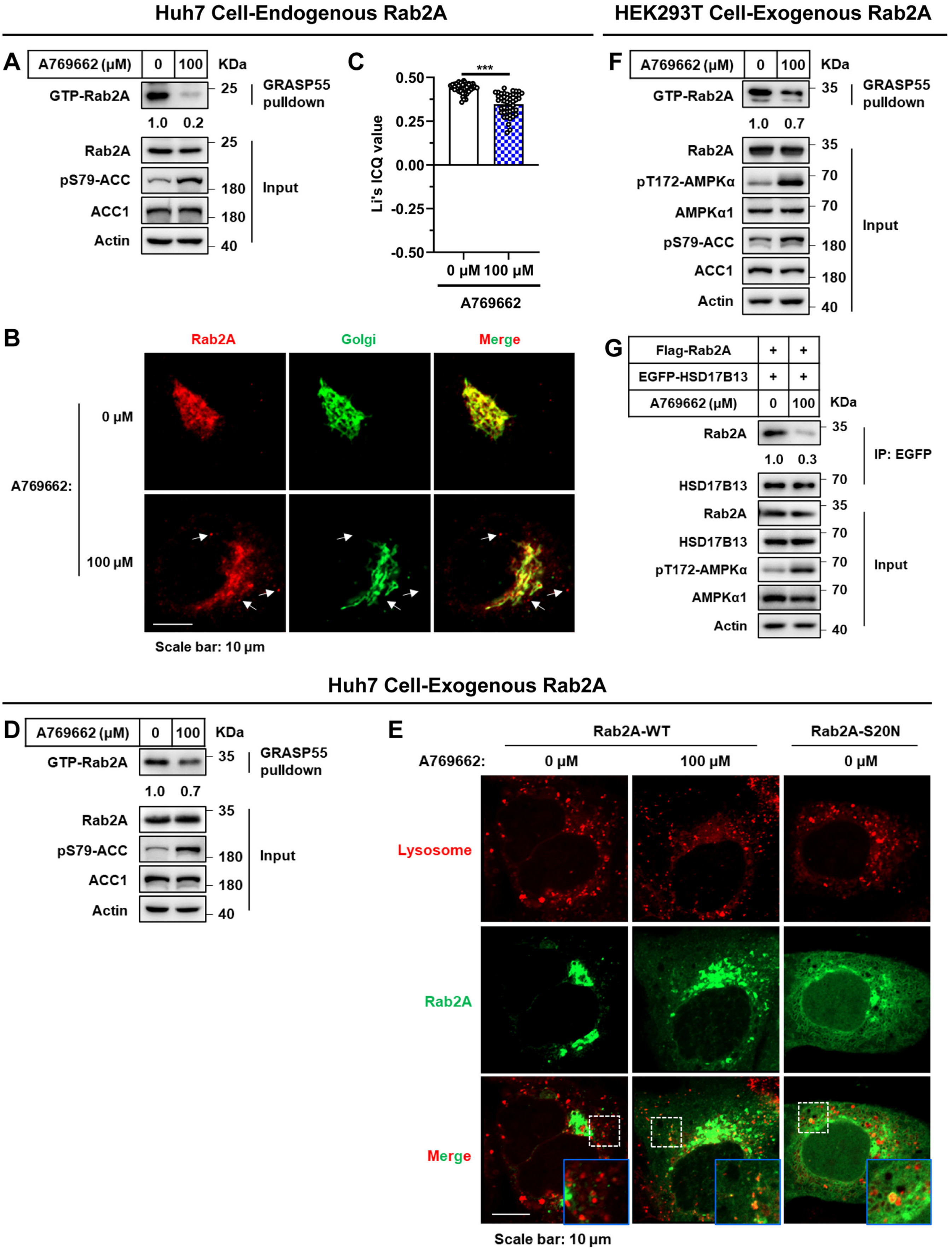
Huh7 Cell-Endogenous Rab2A
HEK293T Cell-Exogenous Rab2A
A
A769662 (μM) 0 100 KDa
GTP-Rab2A 25 GRASP55 pulldown
1.0 0.2
Rab2A 25
pS79-ACC 180 Input
ACC1 180
Actin 40
C
***
Li's ICQ value
0.50
0.25
0.00
-0.25
-0.50
0 μM 100 μM
A769662
F
A769662 (μM) 0 100 KDa
GTP-Rab2A 35 GRASP55 pulldown
1.0 0.7
Rab2A 35
pT172-AMPKα 70
AMPKα1 70 Input
pS79-ACC 180
ACC1 180
Actin 40
B
Rab2A
Golgi
Merge
0 μM
A769662:
100 μM
Scale bar: 10 μm
G
Flag-Rab2A + +
EGFP-HSD17B13 + +
A769662 (μM) 0 100 KDa
Rab2A 35 IP: EGFP
1.0 0.3
HSD17B13 70
Rab2A 35
HSD17B13 70
pT172-AMPKα 70 Input
AMPKα1 70
Actin 40
Huh7 Cell-Exogenous Rab2A
D
A769662 (μM) 0 100 KDa
GTP-Rab2A 35 GRASP55 pulldown
1.0 0.7
Rab2A 35
pS79-ACC 180 Input
ACC1 180
Actin 40
E
Rab2A-WT
Rab2A-S20N
A769662: 0 μM 100 μM 0 μM
Lysosome
Rab2A
Merge
Scale bar: 10 μm

© The Author(s)

Figure EV5. A769662 stimulation attenuates Rab2A activity, Golgi-localization and Rab2A-HSD17B13 binding.

(**A–C**) Huh7 cells were stimulated with A769662 (100 μM), an AMPK agonist, for 4 h in DMEM medium without fetal bovine serum (FBS). Rab2A activity was evaluated using the GRASP55 pulldown assay (**A**). Its subcellular localization was assessed with immunofluorescence (**B**). Endogenous Rab2A was stained with a primary antibody, and the Golgi apparatus was labeled with a primary antibody against GM130. The dispersion of Rab2A signaling was quantified via Li's ICQ value (Li's colocalization value) in Image J ($n = 44$ vs. 44 cells), with white arrows indicating potential signaling away from the Golgi (**C**) ($P < 0.0001$). (**D**, **E**) Huh7 cells, transfected with exogenously expressing Rab2A, were stimulated with A769662 (100 μM) for 4 h in DMEM medium without FBS. The activity of Rab2A was assessed using a GRASP55 pulldown assay and analyzed by Western blotting (**D**). The subcellular localization of Rab2A was evaluated with immunofluorescence, with lysosomes stained using LysoTracker (**E**). (**F**, **G**) HEK293T cells were transfected with exogenously expressing Rab2A plasmids, with or without co-transfection of HSD17B13 plasmids, and subsequently stimulated with A769662 (100 μM) for 4 h in DMEM medium without FBS. Rab2A activity was evaluated using the GRASP55 pulldown assay (**F**). The binding efficiency of Rab2A-HSD17B13 complex was assessed by an EGFP-affinity beads pulldown assay (**G**). Data information: Data in (**C**) are presented as mean ± SEM. Circles in (**C**) correspond to individual cell. *P* value in (**C**) was determined using unpaired two-tailed Student's *t*-test. *** indicates $P < 0.001$.

© The Author(s)
